# Extracellular Loops of the Treponema pallidum FadL Orthologs TP0856 and TP0858 Elicit IgG Antibodies and IgG^+^-Specific B-Cells in the Rabbit Model of Experimental Syphilis

**DOI:** 10.1128/mbio.01639-22

**Published:** 2022-07-12

**Authors:** Kristina N. Delgado, Jairo M. Montezuma-Rusca, Isabel C. Orbe, Melissa J. Caimano, Carson J. La Vake, Amit Luthra, Christopher M. Hennelly, Fredrick N. Nindo, Jacob W. Meyer, Letitia D. Jones, Jonathan B. Parr, Juan C. Salazar, M. Anthony Moody, Justin D. Radolf, Kelly L. Hawley

**Affiliations:** a Department of Medicine, UConn Health, Farmington, Connecticut, USA; b Division of Infectious Diseases, UConn Health, Farmington, Connecticut, USA; c Department of Pediatrics, UConn Health, Farmington, Connecticut, USA; d Department of Molecular Biology and Biophysics, UConn Health, Farmington, Connecticut, USA; e Division of Infectious Diseases, Department of Medicine, and Institute for Global Health and Infectious Diseases, University of North Carolina, Chapel Hill, North Carolina, USA; f Duke Human Vaccine Institute, Durham, North Carolina, USA; g Division of Infectious Diseases and Immunology, Connecticut Children’s, Hartford, Connecticut, USA; h Department of Immunology, UConn Health, Farmington, Connecticut, USA; i Department of Pediatrics, Duke University Medical Center, Durham, North Carolina, USA; j Department of Immunology, Duke University Medical Center, Durham, North Carolina, USA; k Department of Genetics and Genome Sciences, UConn Health, Farmington, Connecticut, USA; University of Oklahoma Health Sciences Center

**Keywords:** *Treponema pallidum*, syphilis, outer membrane protein, extracellular loop, FadL, B cells, vaccine

## Abstract

The resurgence of syphilis in the new millennium has called attention to the importance of a vaccine for global containment strategies. Studies with immune rabbit serum (IRS) indicate that a syphilis vaccine should elicit antibodies (Abs) that promote opsonophagocytosis of treponemes by activated macrophages. The availability of three-dimensional models for Treponema pallidum’s (*Tp*) repertoire of outer membrane proteins (OMPs) provides an architectural framework for identification of candidate vaccinogens with extracellular loops (ECLs) as the targets for protective Abs. Herein, we used Pyrococcus furiosus thioredoxin (*Pf*Trx) as a scaffold to display *Tp* OMP ECLs to interrogate sera and peripheral blood mononuclear cells (PBMCs) from immune rabbits for ECL-specific Abs and B cells. We validated this approach using a *Pf*Trx scaffold presenting ECL4 from BamA, a known opsonic target. Using scaffolds displaying ECLs of the FadL orthologs TP0856 and TP0858, we determined that ECL2 and ECL4 of both proteins are strongly antigenic. Comparison of ELISA and immunoblot results suggested that the *Pf*Trx scaffolds present conformational and linear epitopes. We then used the FadL ECL2 and ECL4 *Pf*Trx constructs as “hooks” to confirm the presence of ECL-specific B cells in PBMCs from immune rabbits. Our results pinpoint immunogenic ECLs of two newly discovered OMPs, while advancing the utility of the rabbit model for circumventing bottlenecks in vaccine development associated with large-scale production of folded OMPs. They also lay the groundwork for production of rabbit monoclonal Abs (MAbs) to characterize potentially protective ECL epitopes at the atomic level.

## INTRODUCTION

The explosive resurgence of syphilis, a sexually transmitted infection caused by the spirochete Treponema pallidum (*Tp*), in the new millennium has fueled a sense of urgency about the need for a vaccine with global efficacy (1, 2). Current conceptions of *Tp* molecular architecture identify the spirochete’s repertoire of rare outer membrane proteins (OMPs) as the principal candidate vaccinogens (3–6). The spirochete’s repertoire of OMPs (the *Tp* OMPeome) consists of two proteins, BamA (TP0326) and LptD (TP0515), involved in OM biogenesis and four paralogous families involved in importation of nutrients or extrusion of noxious substances across the OM: eight-stranded β-barrels, long-chain fatty acid transporters (FadLs), *Tp* repeat proteins (Tprs), and OM factors for efflux pumps (6, 7). Development of OMP-based bacterial vaccines is complicated by the difficulties associated with expressing, purifying, and folding milligram quantities of β-barrel-forming proteins in a conformationally native state (8, 9). As with other diderm bacteria (10, 11), *Tp* OMPs consist of amphiphilic β-barrels with variably sized extracellular loops (ECLs) that bridge adjacent β-strands and extend into the extracellular environment (6, 12, 13). Accurate identification *in silico* of ECL-β-strand boundaries across the *Tp* OMPeome (6) creates the possibility of employing protein engineering strategies to focus the immune response on the critical targets for protective Abs, the ECLs.

The rabbit has long been considered the animal model of choice for investigation of syphilis immunopathogenesis (14–16). Rabbits develop long-lasting immunity to reinfection (14, 15, 17), and it is generally believed that deconvolution of protective responses in the rabbit will inform vaccine development for humans (18). Nevertheless, the outbred nature of the rabbit and the limited commercially available species-specific reagents have proven to be rate limiting for syphilis vaccine research. In recent years, cloning of single B cells from infected individuals has become a powerful strategy for generating neutralizing MAbs against viral pathogens, most notably HIV (19), influenza (20), Zika (21), and SARS-CoV-2 (22). Application of this approach to the rabbit model of syphilis would yield possible new prophylactic agents as well as powerful reagents for atomic level characterization of OMP epitopes associated with spirochete clearance. For this method to be feasible for syphilis, however, one must first identify OMP surface elements that elicit Abs during syphilitic infection and demonstrate the presence of circulating B cells specific for them. Toward these ends, we used Pyrococcus furiosus thioredoxin (*Pf*Trx) scaffolds (23, 24) to detect ECLs in the FadL orthologs TP0856 and TP0858 that elicit strong Ab responses in *Tp*-immune rabbits. We then used the *Pf*Trx-ECL scaffolds as “hooks” to confirm by flow cytometry the presence of rare ECL-specific B cells in peripheral blood mononuclear cells (PBMCs) from immune rabbits. Our results pinpoint immunogenic ECLs of two newly discovered OMPs while advancing the utility of the rabbit model for circumventing bottlenecks in vaccine development associated with large-scale production of folded OMPs. They also lay the groundwork for production of rabbit MAbs to characterize potentially protective ECL epitopes at the atomic level.

## RESULTS

### Generation of *Tp*-immune rabbits.

**FIG 1:**
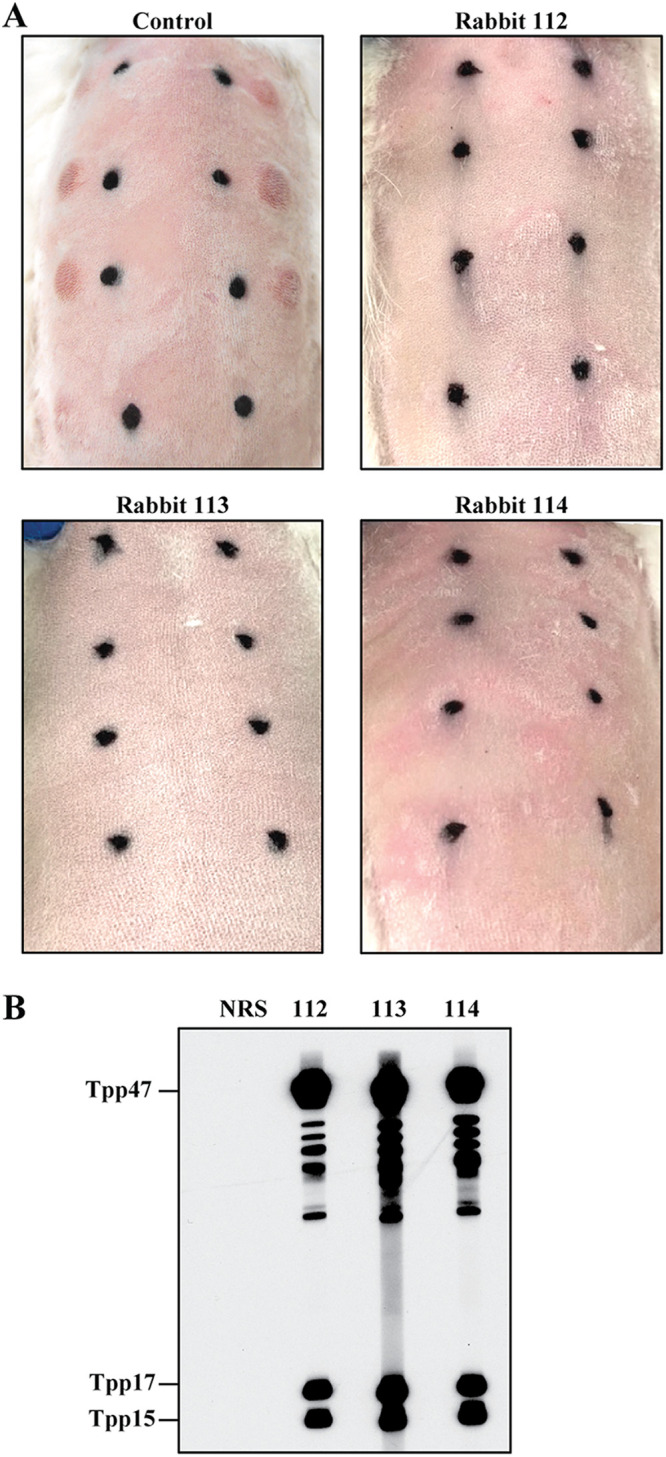
Immune rabbits. (A) Three rabbits inoculated intratesticularly with *Tp* Nichols were challenged 60 days later, along with an uninfected control, on their shaved backs at each of 8 sites with 1 × 10^3^ freshly harvested *Tp.* Black marks indicate location of initial intradermal injections and used to monitoring animals for syphilitic lesion development (representative images 27 days postchallenge). (B) Immunoblot reactivity of sera from the three immune rabbits with *Tp* lysate strips.

Rabbits begin to develop resistance to reinfection with *Tp* several weeks after intratesticular inoculation with ≥1 × 10^7^ organisms and are solidly resistant to intradermal challenge within approximately 2 months (14, 17). To obtain immune sera and PBMCs for the experiments described below, we inoculated three New Zealand White (NZW) rabbits in each testis with 1 × 10^7^ treponemes (Nichols strain) and confirmed their immune status 60 days later by intradermal challenge with 1 × 10^3^ organisms at each of eight sites (Fig. 1A). Immunoblots against *Tp* whole cell lysates revealed that all three rabbits mounted robust Ab responses against multiple treponemal polypeptides (Fig. 1B); particularly strong signals were detected for the 47-, 17-, and 15-kDa lipoprotein immunogens (Tpp47, Tpp17, and Tpp15, respectively) (25–27).

### A Pyrococcus furiosus thioredoxin scaffold to display extracellular loops of *Tp* outer membrane proteins.

**FIG 2:**
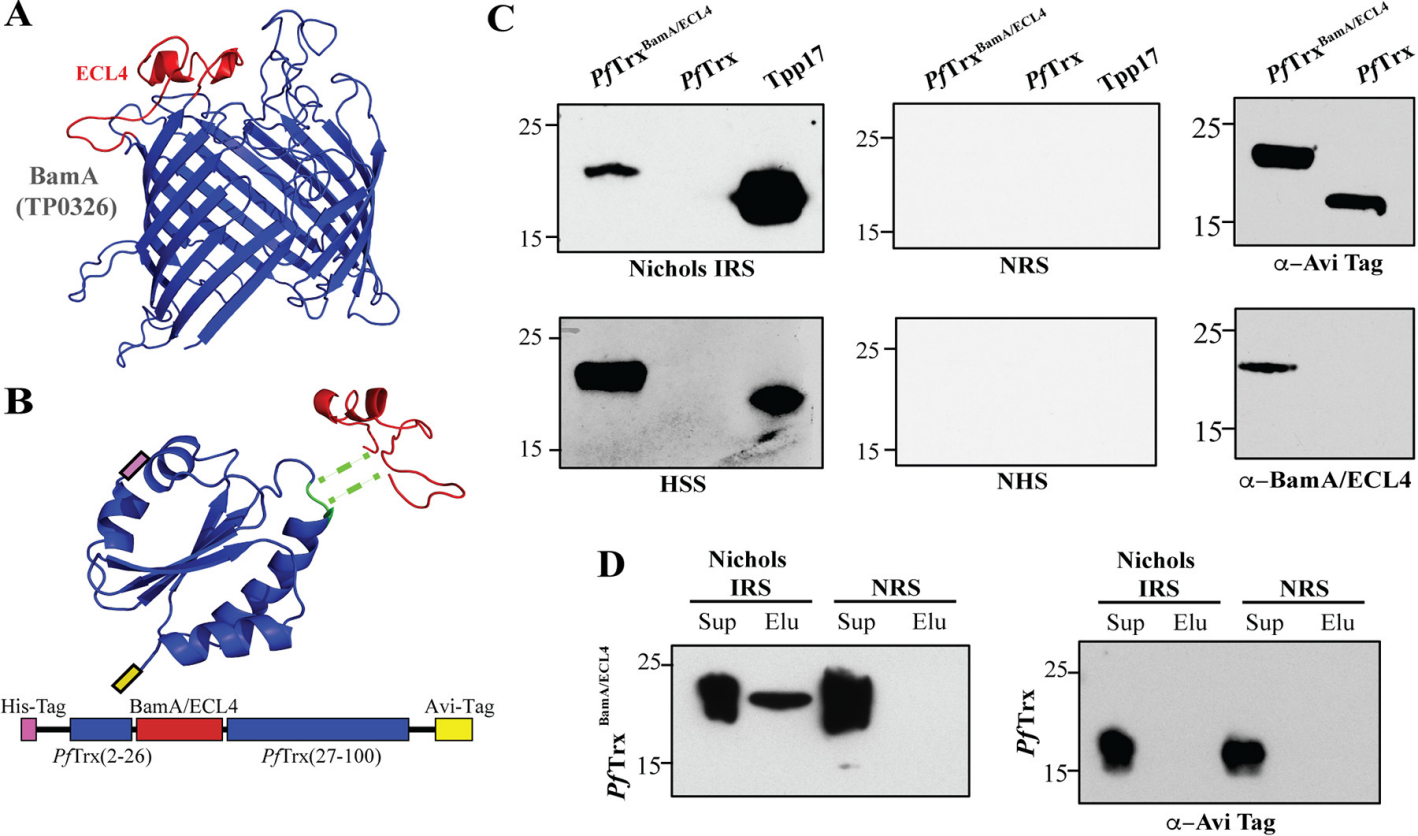
Pyrococcus furiosus thioredoxin (*Pf*Trx) as a scaffold for presentation of extracellular loops (ECLs). (A) ModWeb prediction of the β-barrel of *Tp* BamA (TP0326) (12) with ECL4 shown in red. (B) Phyre2 prediction of the *Pf*Trx structure showing the insertion site (green) for ECLs (here BamA ECL4) and a linear map of the *Pf*Trx^BamA/ECL4^ construct with N-His- and C-Avi- tags shown in pink and yellow, respectively. (C) Reactivity of *Pf*Trx^BamA/ECL4^ and *Pf*Trx with IRS, HSS, NRS, NHS, mouse anti-Avi-Tag, and rat anti-BamA ECL4 antiserum. The highly antigenic lipoprotein, Tpp17, was used as a positive control. (D) Supernatant (Sup) and elution (Elu) fractions from Protein G pull-downs of *Pf*Trx^BamA/ECL4^ and *Pf*Trx with IRS or NRS immunoblotted with Avi-Tag Abs.

We previously identified ECL4 of BamA (TP0326) (Fig. 2A) as an immunodominant ECL and opsonic target (12, 13). To present Nichols BamA ECL4 in a soluble, nondenatured form for Ab binding, we chose *Pf*Trx, which has been used successfully as a scaffold to generate neutralizing Abs against epitopes in the L2 capsid protein of human papillomavirus (23, 24). Compared to the commonly utilized Escherichia coli protein, *Pf*Trx has greater thermal stability, protease resistance, solubility, and little to no cross-reactivity with other thioredoxins (24). We generated a His-tagged version of *Pf*Trx with BamA ECL4 inserted between amino acid residues 26 and 27 along with a C-terminal Avi-Tag for *in vivo* biotinylation (*Pf*Trx^BamA/ECL4^; Fig. 2B and Fig. S1). *Pf*Trx^BamA/ECL4^ purified by Ni-NTA affinity chromatography migrated exclusively as a monomer by size exclusion chromatography (Fig. S1C). Immunoblot analysis revealed that *Pf*Trx^BamA/ECL4^ reacted with Nichols immune rabbit serum (IRS) and human syphilitic serum (HSS), whereas *Pf*Trx alone failed to react with either (Fig. 2C). A pulldown assay was done to evaluate the Ab accessibility of ECL4 presented by *Pf*Trx^BamA/ECL4^. Following overnight incubation of *Pf*Trx^BamA/ECL4^ with IRS or normal rabbit serum (NRS), *Pf*Trx^BamA/ECL4^ was eluted from the beads incubated with IRS but not NRS (Fig. 2D). Importantly, *Pf*Trx was not pulled down with either IRS or NRS, confirming that the IRS-*Pf*Trx^BamA/ECL4^ interaction requires ECL4.

10.1128/mbio.01639-22.3 Text S1 SUPPLEMENTAL MATERIALS AND METHODS. Download TEXT S1, DOCX file, 0.02 MB. Copyright © 2022 Delgado et al. 2022 Delgado et al. https://creativecommons.org/licenses/by/4.0/ This content is distributed under the terms of the Creative Commons Attribution 4.0 International license.

10.1128/mbio.01639-22.1 FIG S1 Purification of *Pf*Trx-ECL constructs, FadLs, OspC, and Tpp17. (A) DNA and corresponding amino acid sequence for the *Pf*Trx scaffold containing BamA ECL4. The codon-optimized gene for *Pf*Trx previously published by Canali et al. (24) is shown in blue. The sequence encoding BamA ECL4 (residues 568-602 of TP0326) is shown in red between residues 26 and 27 of the *Pf*Trx scaffold. The C-terminal Avi-tag for *in vivo* biotinylation is shown in yellow. (B) SDS-PAGE of fractions obtained by Ni-NTA affinity chromatography of *Pf*Trx^BamA/ECL4^ (FT, flow through fraction; A and B: wash fractions); the blue arrow indicates *Pf*Trx^BamA/ECL4^. (C) Size exclusion chromatography of *Pf*Trx^BamA/ECL4^ following Ni-NTA affinity purification. (D) SDS-PAGE of purified TP085 and TP0858, OspC, and Tpp17. (E) SDS-PAGE of *Pf*Trx (lane P) and TP0856 and TP0858 ECL and hatch *Pf*Trx constructs purified by Size exclusion chromatography (lanes 1–7, H). Download FIG S1, TIF file, 2.6 MB. Copyright © 2022 Delgado et al. 2022 Delgado et al. https://creativecommons.org/licenses/by/4.0/ This content is distributed under the terms of the Creative Commons Attribution 4.0 International license.

### The *Tp* FadL orthologs TP0856 and TP0858 harbor immunogenic ECLs.

**FIG 3:**
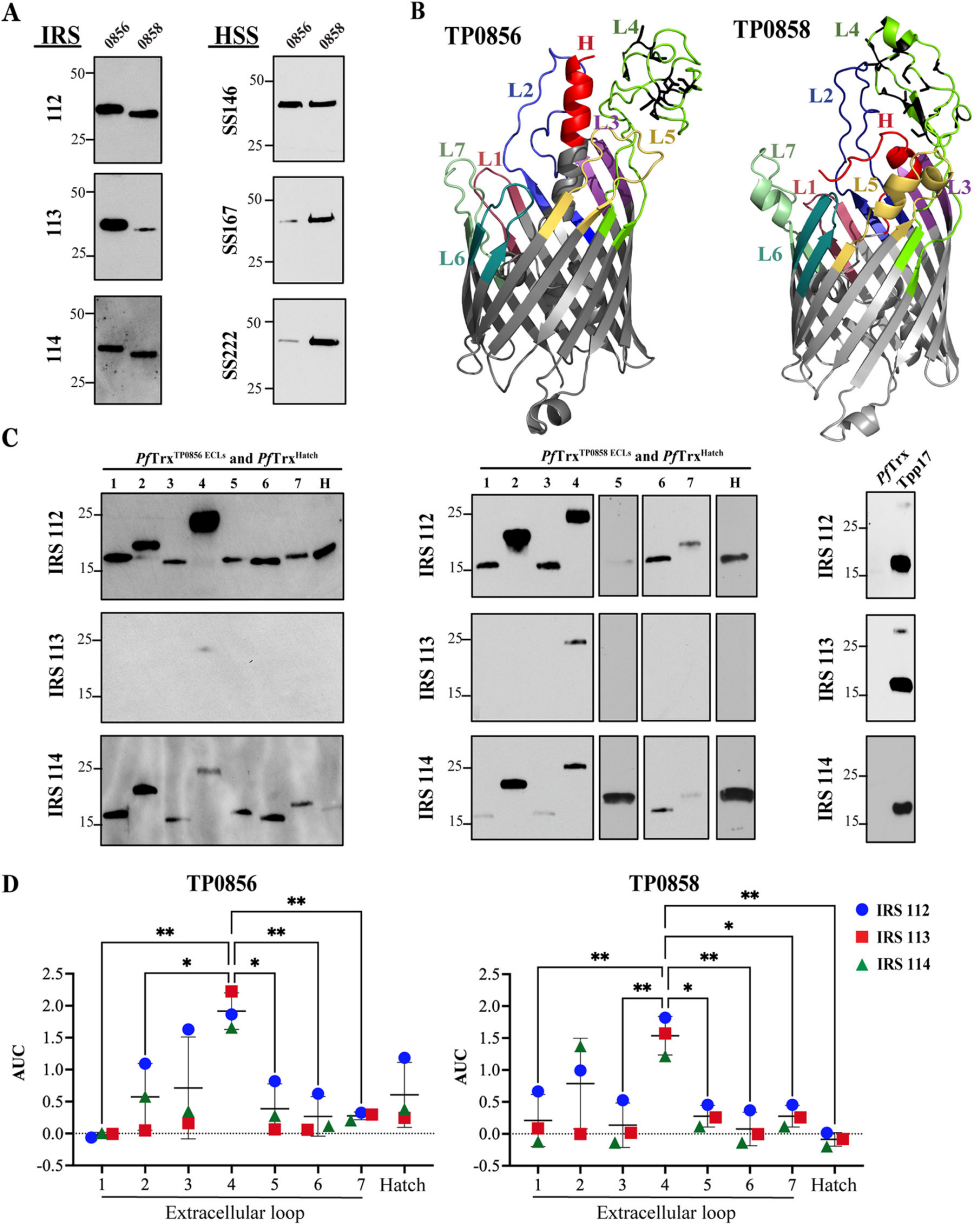
Reactivity of FadL orthologs and extracellular regions of TP0856 and TP0858. (A) Immunoblots of TP0856 and TP0858 against IRS and HSS. (B) trRosetta (89) predictions for the structures of TP0856 and TP0858 (6). The seven ECLs and hatches of each protein are identified; cysteine residues in ECL4 are shown in black. (C) Immunoblots of *Pf*Trx^TP0856/ECL1-ECL7^ and *Pf*Trx^TP0856/Hatch^ (left); *Pf*Trx^TP0858/ECL1-ECL7^ and *Pf*Trx^TP0858/Hatch^ (middle); and *Pf*Trx scaffold and Tpp17 controls (right) against IRS. (D) Reactivity of *Pf*Trx^TP0856/ECL1-ECL7^ and *Pf*Trx^TP0856/Hatch^ (left) and *Pf*Trx^TP0858/ECL1-ECL7^ and *Pf*Trx^TP0858/Hatch^ (right) with IRS, measured as area under the curve (AUC) from ELISA dilutions corrected for *Pf*Trx background. *, *P* ≤ 0.05 or **, *P* ≤ 0.01, significant differences between the means of the groups (respectively) determined by one-way ANOVA with Bonferroni's correction for multiple comparisons.

The *Tp* OMPeome contains five paralogs (TP0548, TP0856, TP0858, TP0859, and TP0865) (6, 7) related to E. coli FadL, a 14-stranded OM fatty acid importer (28). Initially, we sought evidence that FadLs elicit Ab responses during syphilitic infection by examining the immunoblot reactivity of purified TP0856 and TP0858 (both Nichols) with IRS and HSS. IRS from all three immune rabbits reacted strongly with TP0856, whereas HSS showed considerable variability; in contrast, IRS and HSS reacted comparably with TP0858 (Fig. 3A). Collectively, these Ab responses indicate that both proteins are expressed during syphilitic infection in rabbits and humans. We considered the possibility that the variable reactivity of HSS with the TP0856 reflected sequence differences in the infecting strains (Text S1); however, the TP0856 sequences in the three *Tp* clinical strains and the Nichols sequence are identical (Fig. S2A). Interestingly, although sequence differences were observed between TP0858 in the clinical strains and the Nichols protein (Fig. S2B), they did not discernibly impact immunoreactivity of the full-length protein.

10.1128/mbio.01639-22.2 FIG S2 Alignments of TP0856 and TP0858. (A and B) Multiple sequence alignment from clinical isolates of *Tp* for TP0856 and TP0858 using the Nichols sequences for reference. (C) Amino acid sequence alignment of TP0856 and TP0858 indicating predicted β-strands (black arrows), ECLs, and extracellular portions of the hatches (magenta lines). Identical amino acid residues are marked with an asterisk (*); cysteines in ECL4 are marked with red asterisks. Download FIG S2, PDF file, 1.7 MB. Copyright © 2022 Delgado et al. 2022 Delgado et al. https://creativecommons.org/licenses/by/4.0/ This content is distributed under the terms of the Creative Commons Attribution 4.0 International license.

We next used the *Pf*Trx scaffold system to evaluate the immunogenicity of the predicted Ab accessible regions of TP0856 and TP0858. In addition to constructs harboring the seven ECLs from each FadL ortholog, scaffolds also were produced to display the portions of their N-terminal α-helices (“hatches”) predicted to extend through the β-barrel to the extracellular space (Fig. 3B and Fig. S2C) (6). By immunoblotting, IRS 112 and 114 displayed similar antigenic profiles, reacting with multiple *Pf*Trx constructs; particularly noteworthy was their reactivity with ECL2 and ECL4 (Fig. 3C). IRS 113, in contrast, weakly recognized just the ECL4 constructs. Of the three rabbit sera, IRS 112 had the strongest overall reactivity. To enhance the accessibility of epitopes for Ab recognition by enzyme-linked immunoassay (ELISA) (29), wells coated with streptavidin (SP) were used to immobilize the biotinylated *Pf*Trx constructs. By one-way ANOVA, the reactivity of all three IRS with both ECL4s was significantly greater than with the majority of other constructs, although other ECLs (e.g., ECL2 of TP0856 and TP0858) also displayed strong ELISA reactivity (Fig. 3D and Table S1). The strong ELISA reactivity of IRS 113 with both ECL4s was striking given the faint immunoblot results (Fig. 3C). Other discordances between immunoblot and ELISA results also were observed. ECL3, ECL5, and ECL6 of TP0856 reacted weakly by immunoblotting with sera from all three rabbits but strongly by ELISA with sera from rabbit 112. Conversely, IRS 112 and 114 yielded a strong immunoblot signal for ECL1 of TP0856 but reacted poorly by ELISA; similarly, with IRS 114, strong immunoblot but weak ELISA reactivity was observed for ECL5 and the hatch of TP0858 (Fig. 3C and D and Table S1).

10.1128/mbio.01639-22.3 TABLE S1 Summary of area under the curve (AUC) values from ELISA dilution curves. Download Table S1, DOCX file, 0.01 MB. Copyright © 2022 Delgado et al. 2022 Delgado et al. https://creativecommons.org/licenses/by/4.0/ This content is distributed under the terms of the Creative Commons Attribution 4.0 International license.

### Sequences, predicted structures, and electrostatics explain the immunogenic properties of ECL2 and ECL4 in TP0856 and TP0858.

**FIG 4:**
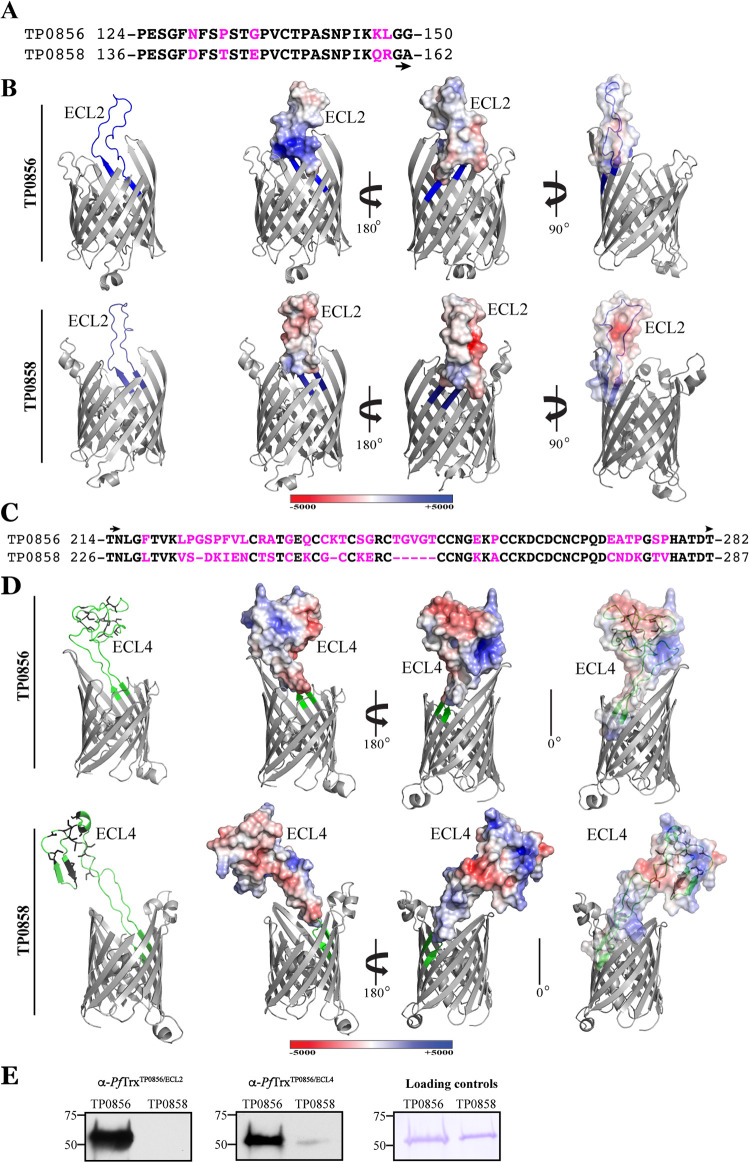
Comparison of sequences, structures and electrostatics of ECL2 and ECL4 of TP0856 and TP858 (Nichols). (A) Alignment of TP0856 and TP0858 ECL2 sequences with substitutions shown in magenta. (B) trRosetta models of TP0856 and TP0858 with ECL2 shown in blue (left). Electrostatics of ECL2s are shown in the same and opposite orientations (middle and right, respectively). (C) Alignment of TP0856 and TP0858 ECL4 sequences with substitutions and deletions shown in magenta. (D) trRosetta models of TP0856 and TP0858 with ECL4 shown in blue (left). Electrostatics of ECL4s are shown in the same and opposite orientations (middle and right, respectively). Some ECLs and the hatches are masked for optimal viewing of electrostatics. (E) Immunoblot reactivity of rabbit anti-*Pf*Trx^TP0856/ECL2^ and anti-*Pf*Trx^TP0856/ECL4^ against full-length TP0856 and TP0858.

The strong immunoreactivities of ECL2 and ECL4 in both TP0856 and TP0858 prompted a detailed analysis of their sequences, predicted structures, and electrostatics. ECL2 is a long, presumably flexible loop consisting of 28 (TP0856) and 26 (TP0858) amino acid residues. Although the amino acid sequences of the ECL2s are highly similar, they contain five nonconservative substitutions (Fig. 4A), three of which (N129→D141, G135→E147, and K147→Q159) markedly alter their surface electrostatics (Fig. 4B) and isoelectric points (pIs of 8.6 and 4.6 for ECL2 of TP0856 and TP0858, respectively). ECL4 is extremely large (60 and 62 residues in TP0856 and TP0858, respectively) and cysteine-rich (12 and 15 cysteines in TP0856 and TP0858, respectively) (Fig. 4C), with the cysteines predicted to cluster distally (Fig. 4D); of note, the high concentration of cysteines is an unusual feature for an OMP ECL. ECL4 of TP0858 is predicted to contain an α-helix and two short antiparallel β-strands absent from its TP0856 counterpart. Comparison of the ECL4 sequences (Fig. 4C) reveals several short stretches of amino acid identity along with numerous nonconservative substitutions that alter charge dispersal (Fig. 4D) but only mildly affect the loop isoelectric points (pIs of 5.0 and 4.7 for TP0856 and TP0858, respectively). For the two ECLs, overlays of electrostatics with the corresponding cartoon diagrams (Fig. 4D, right) reveal obvious differences in the spatial relationships between the charged patches and cysteine clusters.

The above amino acid sequence comparisons predict that ECL2 and ECL4 of TP0856 and TP858 are antigenically distinct. We immunized rabbits with *Pf*Trx^TP0856/ECL2^ or *Pf*Trx^TP0856/ECL4^ and assessed the reactivity of the antisera against the full-length proteins (Fig. 4E). Both antisera strongly recognized TP0856, demonstrating that *Pf*Trx scaffolds can be used to elicit anti-ECL Abs. The *Pf*Trx^TP0856/ECL2^ antiserum exhibited no discernible cross-reactivity with TP0858, while cross-reactivity of the *Pf*Trx^TP0856/ECL4^ antiserum was negligible.

### Comparison between predictive modeling and experimentally derived evidence of B-cell epitopes in TP0856 and TP0858.

**FIG 5:**
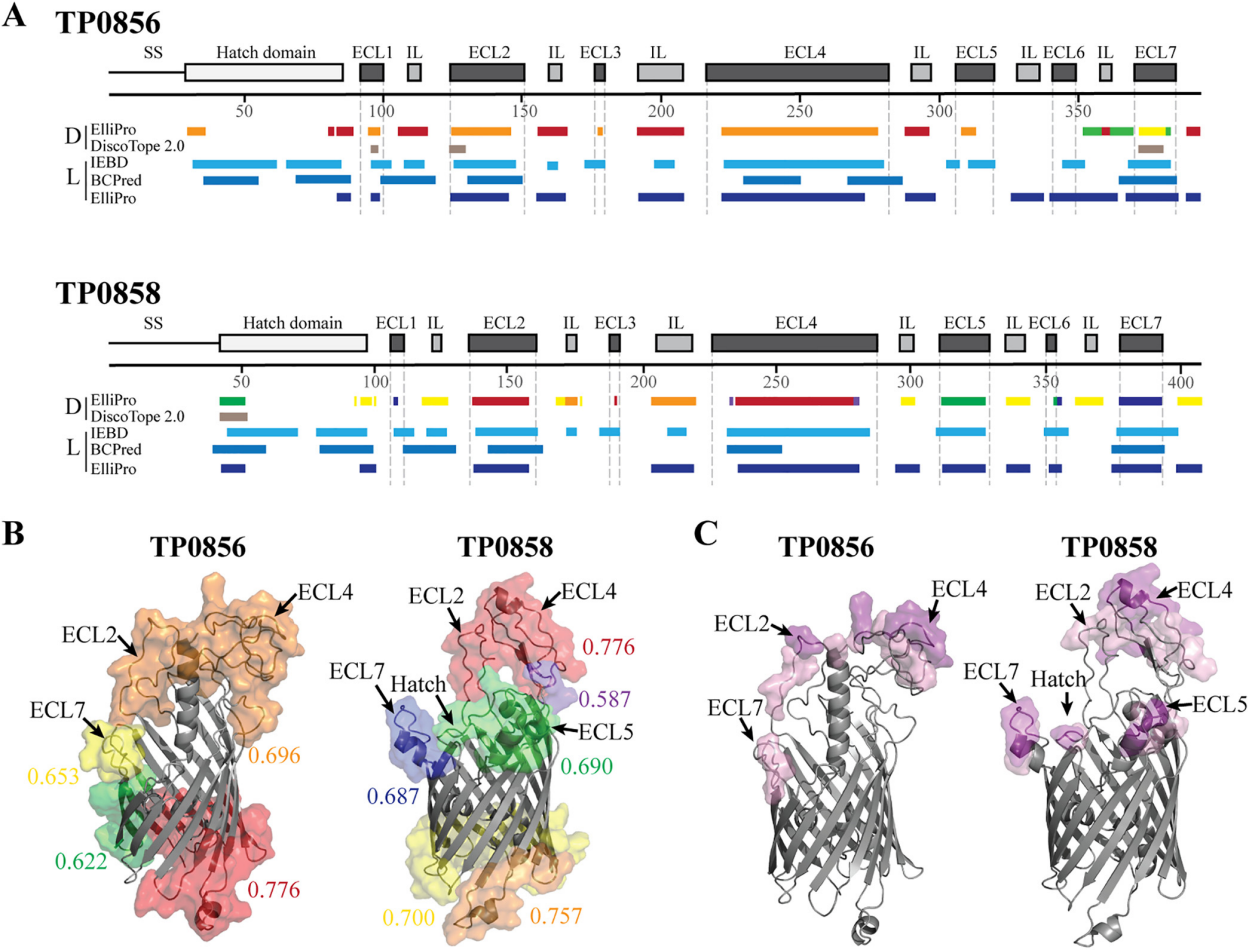
B-cell epitope predictions of TP0856 and TP0858. (A) Cartoon schematics of TP0856 and TP0858 showing the positions of discontinuous (D) and linear (L) BCE predictions using ElliPro (32), DiscoTope 2.0 (31), IEDB (33), and BC pred (34) algorithms. (B) Ribbon diagrams (gray) of TP0856 and TP0858 with discontinuous BCEs predicted by ElliPro (threshold: 0.5) shown as transparent surfaces. Numeric values indicate the average prediction scores for corresponding epitopes. (C) Epitopes with scores ≥0.8 and ≥0.9 are shown as pink and magenta transparent surfaces, respectively.

B-cell epitope (BCE) predictive algorithms are commonly used bioinformatic tools to identify potential targets for antibody-based vaccines (30). The antigenic analyses described above enabled us to compare experimental results for *Tp* OMPs with BCE predictions made using DiscoTope 2.0 (31), ElliPro (32), IEDB (33), and BC pred (34). The availability of structural models for TP0856 and TP0858 (6) enabled prediction of conformational epitopes with DiscoTope 2.0 and ElliPro. For both proteins, there was a reasonable degree of agreement among the four algorithms (Fig. 5A). Although the majority of linear and conformational BCEs map to ECLs and hatches, both proteins contain linear BCEs in periplasmic loops, potentially explaining the immunoblot reactivity of IRS 113 with the full-length proteins (Fig. 3A and C). Consistent with the immunoblot results for IRS 112 and 114, multiple algorithms predicted that ECL2 and ECL4 of TP0856 and TP0858 and ECL7 of TP0856 contain strong linear BCEs. DiscoTope 2.0 predicted that individual ECLs contain conformational epitopes, although the strongest ELISA reactors had either extremely weak scores (ECL2) or scores below threshold (ECL4). Using the default threshold setting (0.5), ElliPro predicted that ECL7 of TP0856 contains a discontinuous BCE; it also identified discontinuous BCEs that span multiple loops on the extracellular and periplasmic faces of both TP0856 and TP0858 (Fig. 5B). To refine the latter predictions for the extracellular regions of the two proteins, we increased the ElliPro thresholds to ≥0.8 and ≥0.9 (Fig. 5C). For TP0856, the increase in threshold to ≥ 0.8 reduced the predicted discontinuous BCEs to three individual loops: ECL2, ECL4, and ECL7. For TP0858, the ≥0.8 threshold yielded separate BCEs consisting of ECL5, ECL7, and the hatch along with an epitope bridging ECL2 and ECL4. The increase in stringency to ≥0.9 further reduced the BCE predications to ECL2 and ECL4 for TP0856 and to ECL4, ECL5, ECL7, and the hatch for TP0858. Overall, the discontinuous BCE predictions correlated well with the ELISA results for ECL2 and ECL4 but not for ECL5 and ECL7.

### Development of a novel gating strategy to identify ECL-specific IgG^+^ B cells.

**FIG 6:**
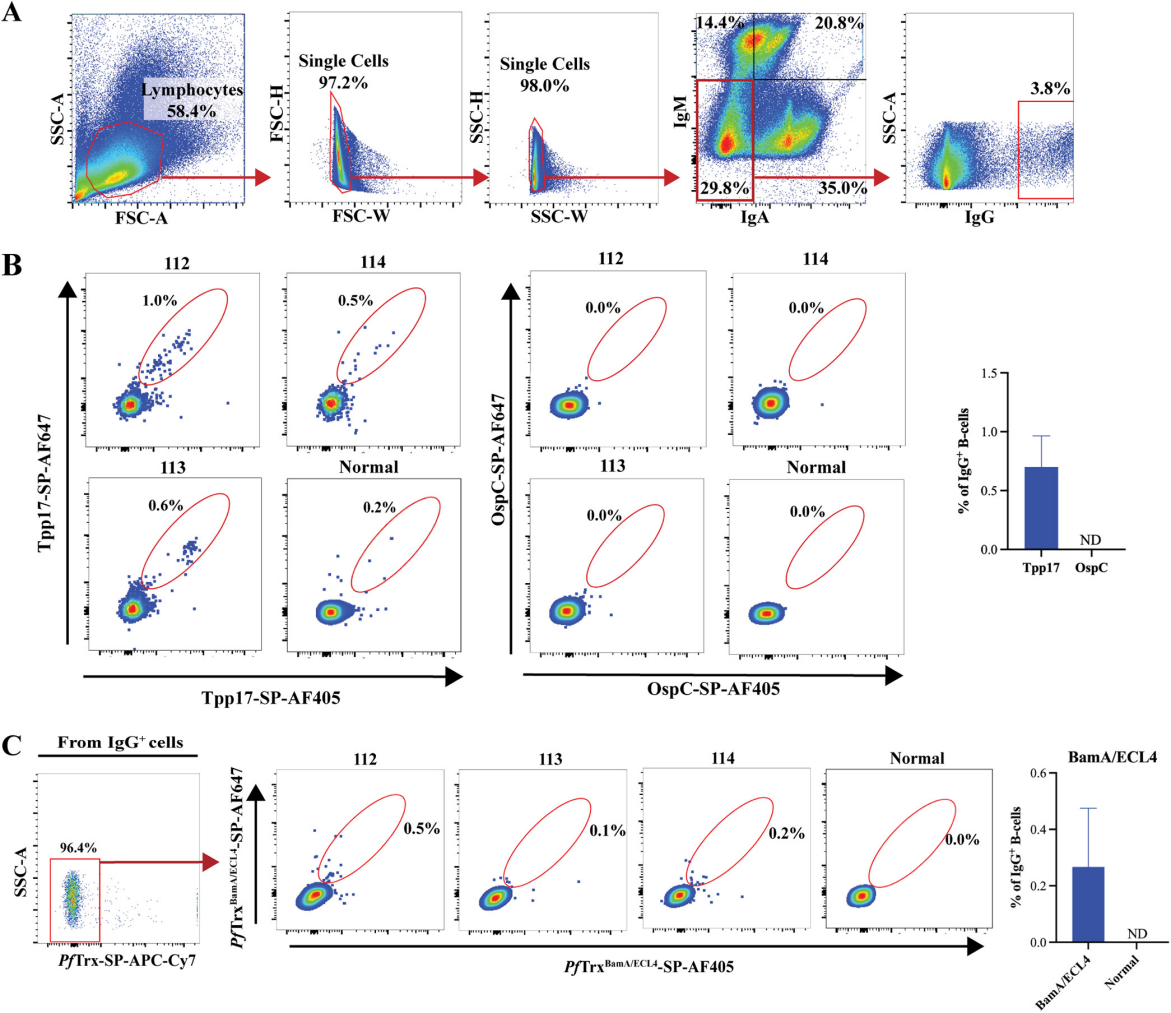
Identification of Tpp17-specific rabbit IgG^+^ B cells. (A) Gating strategy: After gating lymphocytes, doublets were excluded in FSC-H and -W, and SSC-H and -W plots. IgG^+^ cells were identified from the IgM and IgA double-negative cells. (B) Tpp17-specific B cells were gated from IgG^+^ cells as double-positive cells for Tpp17-SP-AF405 and Tpp17-SP-AF647. B. burgdorferi OspC conjugated to SP-AF405 and SP-AF647 was used as a negative control. The mean frequencies of antigen-specific IgG^+^ B cells are shown in the accompanying bar graphs. (C) Identification of IgG^+^ B cells specific for *Pf*Trx^BamA/ECL4^ using the gating strategy shown in panel A. IgG^+^ *Pf*Trx^+^ cells were excluded using *Pf*Trx-SP-APC-Cy7. From each immune rabbit, B cells specific for *Pf*Trx^BamA/ECL4^ were identified within the IgG^+^ *Pf*Trx^Neg^ gate as cells double-positive for SP-AF405 and SP-AF647; PBMCs from a normal rabbit were used as a control. The mean frequencies of IgG^+^ B cells specific BamA ECL4 are shown in the accompanying bar graphs.

Two previous reports have described flow cytometric identification of IgG^+^ antigen-specific B cells in immunized rabbits (35, 36). In these studies, however, the anti-rabbit IgG conjugates used appeared to cross-react with B cells expressing IgM, and the panels did not include reagents to stain for B cells expressing IgA, an isotype highly abundant in rabbits (37). To set the stage for future generation of MAbs directed against immunoreactive ECLs, we developed a novel gating strategy to detect rare ECL-specific B cells in PBMCs from syphilis immune rabbits. To improve the accuracy of selection of IgG^+^ B cells, we made the following modifications to prior staining panels (Fig. 6A and Table S2): (i) use of an IgM-FITC conjugate that in preliminary experiments produced less background than conjugates used in the prior studies; (ii) identification of a commercially available anti-rabbit IgG without IgM cross-reactivity, and (iii) addition of an anti-rabbit IgA conjugate. To enhance sensitivity for detection of specific IgG^+^ B cells, we expressed antigens with a C-terminal Avi-Tag to allow for site-specific *in vivo* biotinylation (38). To enhance specificity, in all experiments PBMCs were probed with antigen tetramers separately labeled with either SP-AF647 or SP-AF405; only double-positive B cells were considered antigen-specific. We initially validated this system by probing PBMCs with the strongly antigenic lipoprotein Tpp17 (Fig. 6A and B). Tpp17-specific cells were detected at frequencies of 1.0%, 0.6%, and 0.5% within the IgG^+^ gates for rabbits 112, 113, and 114, respectively (Fig. 6B). IgG^+^ cells from the three immune rabbits did not bind biotinylated OspC, an immunogenic lipoprotein required for mammalian infection by Borrelia burgdorferi, the Lyme disease spirochete (39, 40) (Fig. 6B).

10.1128/mbio.01639-22.4 TABLE S2 Summary of antibody reagents used for identification of IgG+ antigen-specific rabbit B cells. Download Table S2, DOCX file, 0.01 MB. Copyright © 2022 Delgado et al. 2022 Delgado et al. https://creativecommons.org/licenses/by/4.0/ This content is distributed under the terms of the Creative Commons Attribution 4.0 International license.

We next assessed the utility of the *Pf*Trx scaffolds for detection of B cells specific for antigenic ECLs. Employing the same gating strategy described in Fig. 6A, we probed immune rabbit PMBCs with *Pf*Trx^BamA/ECL4^-SP-AF647, *Pf*Trx^BamA/ECL4^-SP-AF405, and *Pf*Trx conjugated to APC-Cy7. BamA ECL4-specific B cells were identified as double-positive for SP-AF647 and SP-AF405 after excluding cells with nonspecific binding to APC-Cy7-*Pf*Trx. The frequencies of BamA ECL4-specific B cells were 0.5%, 0.1%, and 0.2% for immune rabbits 112, 113, and 114, respectively (Fig. 6C).

### Syphilis in rabbits elicits B cells specific for ECL2 and ECL4 of TP0856 and TP0858.

**FIG 7:**
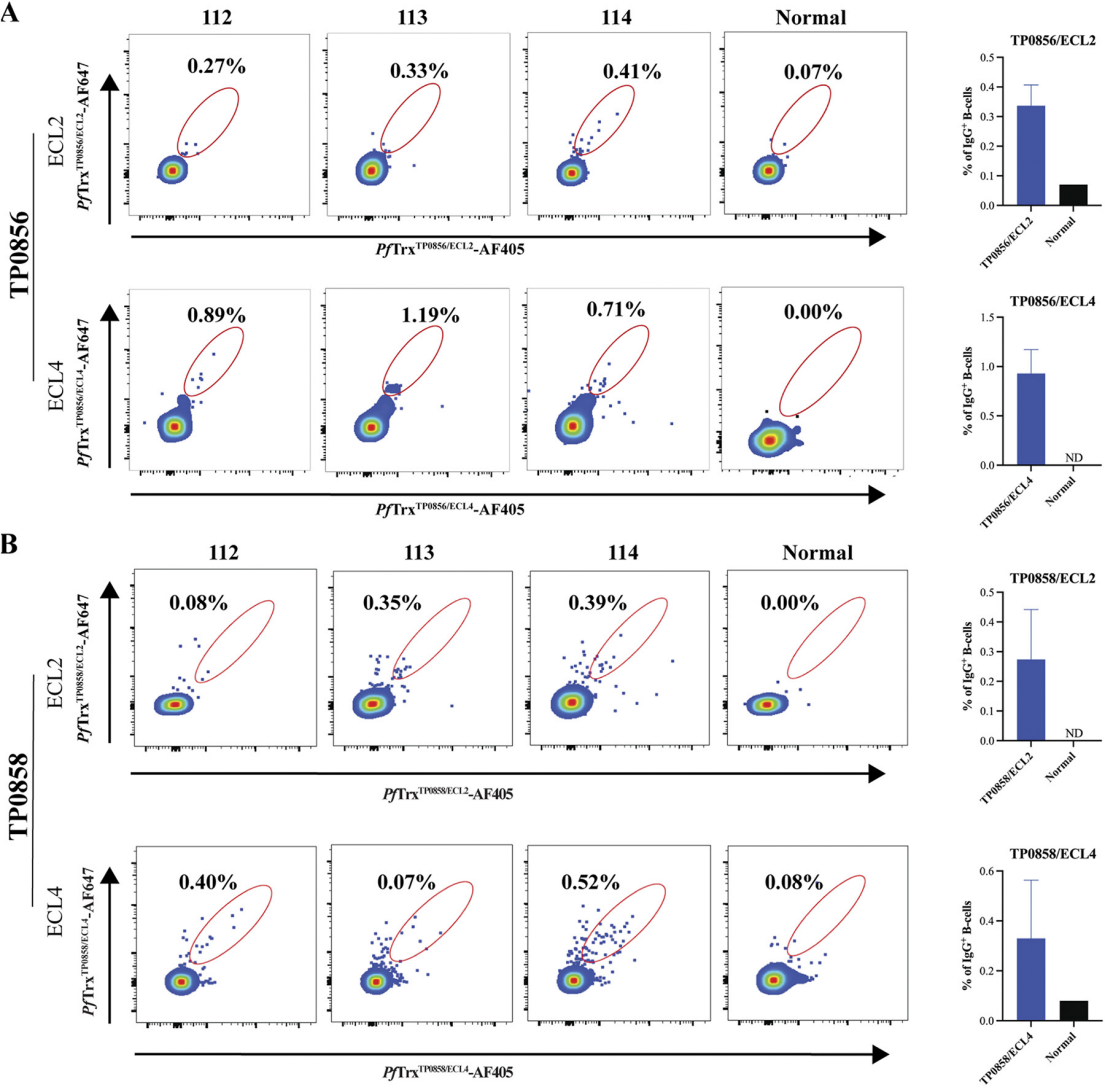
Identification of circulating B cells specific for ECL2 and ECL4 of TP0856 and TP0858. IgG^+^ B cells specific for ECL2 and ECL4 in TP0856 (A) and TP0858 (B) were identified within the IgG^+^ *Pf*Trx^Neg^ gate as cells double positive for SP-AF405 and SP-AF647; PBMCs from a normal rabbit were used as a control. The mean frequencies of the individual ECL-specific IgG^+^ B cells are shown in the accompanying bar graphs.

Lastly, we interrogated PBMCs from the three immune rabbits with the biotinylated FadL ECL2 and ECL4 *Pf*Trx constructs. As shown in Fig. 7, we detected B cells specific for ECL2 and ECL4 of TP0856 and TP0858 at varying frequencies in all three rabbits; the mean frequencies were in a similar range to those for Tpp17 and BamA ECL4. For TP0856 (Fig. 7A), the mean frequency of ECL4-specific cells (0.93%) was approximately three times higher than that of ECL2-specific cells (0.34%); in general, these results agree with the greater ELISA reactivities observed for ECL4. With rabbits 112 and 114, the detection of ECL2- and ECL4-specific B cells for TP0856 was in line with their reactivity by immunoblot and ELISA. With rabbit 113, the results for TP0856 were less straightforward. Its B cells recognized ECL2 despite the lack of Ab reactivity by immunoblot or ELISA. Surprisingly, it exhibited greater B cell reactivity for ECL4 than either rabbit 112 or 114. For TP0858 (Fig. 7B), the mean frequencies of ECL2- and ECL4-specific B cells were similar, although lower overall than for TP0856. Correlations between B-cell frequencies and Ab reactivity were less evident for TP0858. Rabbit 114 exhibited B-cell reactivity for ECL2 in concert with strong Ab reactivity by both immunoblot and ELISA, while rabbit 112 exhibited modest B-cell recognition for ECL2 but strong immunoblot and ELISA reactivity. Rabbit 113 displayed a low B-cell frequency for ECL4 with a strong ELISA and weak immunoblot.

## DISCUSSION

An issue of fundamental importance to syphilis vaccine research is the nature of protection afforded by infection with *Tp* (41, 42). Assessing immunity in humans is complicated by treatment, which aborts the immune response during early syphilis (43, 44), and still poorly understood differences in OMP repertoires between *Tp* strains currently in circulation (45, 46). The rabbit model has provided definitive evidence for immunity to homologous rechallenge (14, 15, 17), and it is widely believed that deconvolution of this protective response will inform vaccine development for humans. The current conception of protective immunity is that spirochete clearance is driven by opsonophagocytosis and that production of so-called “functional” Abs must be paired with cellular responses to activate professional phagocytes, particularly macrophages (47–49). Historically, immune rabbit serum has been a “black box” that contains opsonic Abs against unknown targets on the *Tp* surface. Herein, we exploited our structural modeling of the *Tp* OMPeome (6) with an eye toward strategies for vaccine development that avoid the bottlenecks associated with full-length OMPs.

Data obtained by epitope mapping of proteins with overlapping peptides are limited to linear epitopes and devoid of structural context for the protein as a whole. The availability of structural information for an OMP enables one to focus on the regions of the protein, namely, ECLs, relevant to Ab-mediated clearance (11, 50, 51). There is compelling evidence that unfolded recombinant OMPs have poor protective capacity against their respective pathogens (52, 53), indicating that conformational epitopes are critical for immunity. ECLs can be fixed with stable conformations as a result of interactions with the barrel or with each other (51, 54), while many are mobile and flexible (55–57). Structural characterization of ECL-Ab complexes reveals that even mobile ECLs adopt specific conformations when bound by bactericidal Abs (58). Use of a scaffold that presents ECLs as tethered peptides facilitates presentation in a form that conformationally mimics the native immunogen. Researchers have engineered multiple scaffolds (e.g., cyclic peptides, virus-like particles [VLPs], *Pf*Trx, TbpB, and ferritin) to present tethered peptide antigens (23, 24, 59–62); to date, their utility for OMP ECLs has not been extensively studied. Several considerations, in addition to thermal stability and ease of purification, led to our selection of *Pf*Trx for display of *Tp* OMP ECLs: (i) *Pf*Trx-peptide chimeras are abundantly expressed in E. coli as soluble proteins; (ii) *Pf*Trx chimeras with HPV major capsid protein L2 peptides (e.g., residues 20 to 38) induced neutralizing Abs, implying conservation of conformational epitopes (24); (iii) unlike some scaffolds (e.g., VLPs) (60), *Pf*Trx can accommodate ECLs of various lengths while retaining solubility (23); and (iv) *Pf*Trx chimeras can be heptamerized to increase epitope density (23). The versatility of the *Pf*Trx scaffold was attractive because modeling predicts that the *Tp* OMPeome contains ECLs with a wide spectrum of sizes, including large ECLs with secondary structural elements (6, 12, 13). The addition of a C-terminal Avi-Tag allowed for site-directed biotinylation *in vivo*, enhancing presentation of the ECL in ELISAs using SP for antigen capture (63) as well as optimizing tetramer formation for flow cytometry. We used BamA ECL4 to validate the *Pf*Trx system given our previous work demonstrating that this loop is immunogenic in humans and rabbits with syphilis as well as an opsonic target (12, 13). Besides confirming that Abs in IRS recognized *Pf*Trx^BamA/ECL4^ in both denatured and undenatured forms, we demonstrated by pulldown that *Pf*Trx displays ECL on its surface, ensuring its suitability for subsequent flow cytometry studies to identify specific B cells.

Mining of the *Tp* genome for exported β-barrel forming proteins revealed that the bacterium contains several families of OMPs that collectively enable this extreme auxotroph to import the wide array of substances needed to survive in its obligate human host (4, 6, 45, 64). The FadLs comprise a family of five 14-stranded β-barrels with hydrophobic channels for uptake of fatty acids and other poorly soluble nutrients (e.g., flavins) (28, 65, 66). Homology models of TP0856 and TP0858 predict two large ECLs (>20 amino acids) in each (6). The largest (ECL4) contains a hydrophobic cleft postulated to serve as a conduit for directing substrates toward the barrel interior. An unusual feature of FadL proteins is the hatch that plugs the lumen of the barrel, regulating traversal of the channel by substrates *en route* to the periplasm (28). Unlike E. coli’s FadL (28, 65), the hatches of TP0856 and TP0858 are predicted to extend into the external milieu (6). These external features, presumably indispensable for transport function, become potential Achilles’s heels for the spirochete from the standpoint of protective immunity.

We began our analysis of the FadLs by confirming that the full-length proteins are immunogenic during infection. Rabbits are known to be capable of generating extremely robust humoral responses (67). While rabbits and humans produced anti-FadL Abs, the rabbit response appears to be stronger, a finding we have observed with other OMPs (12, 68, 69). The potency of anti-OMP responses mounted by rabbits may explain why they clear *Tp* so efficiently following inocula many-fold greater than those transmitted among humans during sexual activity (16, 47, 70). The ability of rabbits to mount strong Ab responses against *Tp* OMPs also speaks to the utility of the rabbit model as a screen for candidate vaccinogens. Sequence and antigenic variability among bacterial OMP ECLs (50, 51), including those of *Tp* (12, 13, 46), is well recognized. This variability could complicate analysis of Ab responses in human populations in which multiple strains of the spirochete and, therefore, multiple variants of individual OMPs, might be circulating. Thus, a particular advantage of the experimental rabbit model is that the OMP sequences of the infecting strain are known. It is noteworthy that rabbit 113 mounted the weakest overall Ab response to the FadL ECLs but was no less protected against challenge. Conceivably, Ab responses against other OMPs not investigated herein explain this intriguing observation. If so, variable anti-OMP responses by outbred immune rabbits could inform future vaccine studies by pointing to different combinations of OMPs capable of inducing a protective response.

A comparison of experimental results and BCE predictions revealed that BCE-predictive algorithms can be helpful for interpreting antigenicity data and pinpointing potential protective targets. BCE algorithms predicted that TP0856 and TP0858 harbor epitopes distributed along their lengths, including Ab inaccessible regions. These predictions also underscore the importance of focusing antigenic analyses on surface-exposed regions of the proteins. For both proteins, ECL2 and ECL4 were the most antigenic loops by immunoblot and/or ELISA. TP0856 and TP0858 are more closely related to each other than to the other FadLs (6). Examination of the respective ECL2 and ECL4 sequences suggested that the respective loops would be antigenically distinct. Antisera raised using *Pf*Trx^TP0856/ECL2^ and *Pf*Trx^TP0856/ECL4^ confirmed that there is essentially no cross-reactivity between ECL2 and ECL4 dyads; one can infer from these results that Abs and B cells in immune rabbits are recognizing distinct epitopes on each ECL. This antigenic dichotomy works to the spirochete’s advantage, since responses against one protein will not target the other but is disadvantageous from a vaccine standpoint because two proteins will be needed as immunogens to generate Ab responses targeting both.

The discordant immunoblot and ELISA reactivity of IRS 113 with *Pf*Trx^TP0856/ECL4^ and *Pf*Trx^TP0858/ECL4^ stood apart from the other two immune sera, which reacted well in both formats. These results strongly suggest that (i) ECL4 contains conformational as well as linear epitopes, (ii) the *Pf*Trx construct can display both, and (iii) rabbit 113 produced Abs predominantly against the former. The BCE predictions further support that ECL4 harbors both linear and conformational epitopes. Structural analysis of ECL4 revealed features consistent with this notion. Most notable is the unusual cluster of cysteine residues at the distal end that when disulfide-bonded would create a rigid charged structure even if the entire ECL is mobile. ECL2 has a different reactivity profile than ECL4: only two rabbits produced Abs against it, and both immune sera reacted well with *Pf*Trx^TP0856/ECL2^ and *Pf*Trx^TP0858/ECL2^ by immunoblot and ELISA. Like ECL4, ECL2 is predicted to contain strong linear and conformational BCEs. Strong linear epitopes are not unexpected given the loop’s size and electrostatics. Unlike ECL4, conformational epitopes in ECL2 are not readily discernible given that the ELISA results could reflect reactivity with linear and/or conformational epitopes. Other ECLs (ECL3, ECL5, and ECL6 of TP0856) also displayed discordant ELISA and immunoblot results, suggesting that conformational epitopes are not limited to ECL2 and ECL4. Interestingly, one BCE algorithm (Ellipro [32]) predicted that in TP0858 contiguous regions of ECL2 and ECL4 form a discrete epitope, implying that not all ECL epitopes can be replicated with single loop constructs. Structural characterization of TP0858 is needed to assess the spatial and physical relationships between these two large loops. It is noteworthy that some small ECLs (e.g., ECL5 of TP0858 and ECL7 of TP0856 and TP0858) were predicted to contain both linear and conformational epitopes but were reactive only by immunoblot. There are two possible explanations for these discordances. One is that the prediction of conformational epitopes is erroneous. Another is that the linear epitopes detected by immunoblot were masked in the undenatured *Pf*Trx construct. Distinguishing between these possibilities will require using a scaffold that tightly constrains small ECLs as opposed to the *Pf*Trx system used herein in which ECLs are inserted into a flexible region of the scaffold.

Application of the “learning from nature” concept for vaccine design requires knowledge of the bacterial antigens that elicit protective Abs during infection (71). In the case of syphilis, we currently have little information as to which OMPs induce Abs, no less protective responses, and it is not feasible to express and refold every member of the OMPeome to identify those with protective capacity. Given *Tp*’s well-deserved reputation for “stealth pathogenicity” due to its poor surface reactivity (4, 45, 72), it is also likely that some OMPs fail to elicit Ab responses. The assumption that a cocktail of antigens will be required adds an additional layer of complexity to the search for protective OMP vaccinogens (73). In this study, we demonstrated the feasibility of using a single scaffold to screen IRS for ECL Abs and generate robust loop-specific antisera for future surface localization and opsonophagocytosis assays. We also validated the use of *Pf*Trx constructs as hooks for detection of cognate rare circulating B cells. On the whole, the observed B-cell frequencies were similar to rare B-cell populations detected in viral infections of humans (74) and nonhuman primates (75), although confirmation of the specificity of the B cells detected awaits generation of ECL-specific monoclonal Abs. We note, however, that antigenicity did not always correlate precisely with B-cell frequency; this could reflect the fact that ECL-specific Abs could be generated by Ab secreting cells that reside in tissues other than peripheral blood such plasmablasts or terminally differentiated plasma cells (76, 77). However, antigen-specific B-cell populations in later stages of differentiation are difficult to characterize by flow cytometry due to the paucity of B-cell surface markers including immunoglobulin (78). Because of their propensity for strong Ab responses, in recent years, rabbits have gained favor as a source of MAbs, and the technology for producing rabbit MAbs has become well established (79). The availability of ECL-specific MAbs would enable characterization of opsonic epitopes at the atomic level. This structure-based approach, coupled with identification of conserved, immunogenic loops based on genomic sequences of geographically diverse *Tp* strains (13, 80–82), could eventually pave the way for development of a broadly protective, multi-ECL syphilis vaccine.

## MATERIALS AND METHODS

### Ethics statement.

Animal experimentation was conducted following the *Guide for the Care and Use of Laboratory Animals* (8th edition) in accordance with protocols reviewed and approved by the UConn Health Institutional Animal Care and Use Committee under the auspices of Animal Welfare Assurance A3471-01. Following informed consent, sera were obtained from untreated, HIV seronegative individuals with secondary syphilis at Centro Internacional de Entrenamiento e Investigaciones Médicas (CIDEIM), in Cali, Colombia (83, 84). The study protocol and consent form were approved by the human subjects board at CIDEIM.

### Propagation of *Tp* Nichols and generation of immune rabbits.

The Nichols strain of *Tp* was propagated by intratesticular inoculation of adult male New Zealand White (NZW) rabbits as previously described (4, 49). Immune rabbits were generated by inoculation of three rapid plasma reagin nonreactive adult NZW rabbits in each testis with 1 × 10^7^ treponemes in 500 μL CMRL containing 20% NRS. Sixty days postinoculation, the immune status of the animals was confirmed by intradermal challenge with 1 × 10^3^ freshly extracted *Tp* Nichols at each of eight sites on their shaved backs; a nonimmune rabbit was used as a control.

### Structural models.

The homology model of BamA (TP0326) was generated by comparative modeling using the solved crystal structure of BamA of Neisseria gonorrhoeae (PDB accession no. 4K3B) as previously described (12). Models for TP0856 and TP0858 were generated using trRosetta as previously described (6). PyMOL (85) was used to identify the extracellular regions of BamA, TP0856, and TP0858. The electrostatic potentials of ECL2 and ECL4 of TP0856 and TP0858 were calculated using the adaptive Poisson-Boltzmann solver (APBS) (86). The model for *Pf*Trx was generated using the Phyre2 server (87).

### Cloning of His-tagged recombinant proteins.

Oligonucleotide primers (Table S3) were purchased from Sigma-Aldrich. *Tp* Nichols DNA was extracted from spirochetes using the QIAamp DNA minikit (Qiagen), eluted in 100 μL of elution buffer at 70°C, and stored at −20°C. Tpp17 (TP0435) lacking its signal sequence was PCR-amplified and ligated into NdeI-XhoI digested p28BIOH-LIC (Addgene plasmid 62352). TP0856 lacking its signal sequence was amplified from *Tp* Nichols genomic DNA. TP0858 lacking its signal sequence was PCR-amplified from the full-length, codon-optimized synthetic gene (GenScript). Gel-purified amplicons for TP0856 and TP0858 were cloned into NdeI-XhoI digested pET28a vector (Novagen) by In-Fusion cloning (TaKaRa).

10.1128/mbio.01639-22.5 TABLE S3 Primers. Download Table S3, DOCX file, 0.03 MB. Copyright © 2022 Delgado et al. 2022 Delgado et al. https://creativecommons.org/licenses/by/4.0/ This content is distributed under the terms of the Creative Commons Attribution 4.0 International license.

### Cloning of *Pf*Trx^ECL^ scaffolds.

See Table S3 for oligonucleotide primers. A codon-optimized version of *Pf*Trx (24) with *Tp* BamA ECL4 inserted between amino acid residues 26 and 27 of the native *Pf*Trx and a C-terminal Avi-Tag (GLNDIFEAQKIEWHE) (Fig. S1) was synthesized by Genewiz. The resulting construct (*Pf*Trx^BamA/ECL4^) was PCR amplified and cloned into NdeI-XhoI digested pET28a by In-Fusion cloning. To generate an “empty” *Pf*Trx scaffold (pET28a*^Pf^*^Trx^; Addgene plasmid 181882), *Pf*Trx^BamA/ECL4^ was digested with BamHI to remove ECL4 and then self-ligated. *Pf*Trx constructs for ECLs 1, 3, and 5 to 7 and the hatch regions of TP0856 and TP0858 were generated by inverse PCR of pET28a*^Pf^*^Trx^ using primers containing the corresponding ECL or hatch coding sequences followed by InFusion cloning. *Pf*Trx constructs containing ECL2 and ECL4 from *tp0856* and *tp0858* were generated by PCR-amplifying the loops from codon-optimized synthetic genes followed by insertion into BamHI-digested pET28a*^Pf^*^Trx^ by InFusion cloning.

### Cloning of Borrelia burgdorferi *ospC*-AviTag.

*ospC-*AviTag was generated by replacing the coding sequence for *Pf*Trx in pET28a*^Pf^*^Trx^ with the *ospC* gene (*bbb19*, Borrelia burgdorferi B31) lacking its signal sequence.

### Expression and purification of recombinant proteins.

TP0856 and TP0858 were expressed in E. coli Overexpress C41(DE3) (5). Cells were centrifuged at 6,000 × *g* for 15 min at 4°C, resuspended in buffer (50 mM Tris [pH 7.5], 50 mM NaCl) containing 100 μg of lysozyme and protease inhibitors, lysed by sonication, and then centrifuged at 20,000 × *g* for 30 min at 4°C. Recombinant proteins were solubilized in 50 mM Tris (pH 7.5), 100 mM NaCl, and 6 M GuHCl for 60 min at 25°C. After centrifugation at 20,000 × *g* for 30 min at 4°C, the solubilized proteins were purified over Ni-NTA resin using 50 mM Tris (pH 7.5), 200 mM NaCl, 40 mM imidazole, and 8 M urea and eluted using 300 mM imidazole. Proteins were concentrated using Amicon Ultra filter (10 kDa cut-off), flash-frozen, and stored at −80°C.

*Pf*Trx proteins were expressed in E. coli BL21(DE3) transformed with BirA (BPS Bioscience) (88) for *in vivo* biotinylation; transformants were grown in LB-Miller broth containing 50 μg/mL kanamycin and spectinomycin and 50 μM d-biotin. Following overnight induction with 1 mM isopropyl β-d-1-thiogalactopyranoside (IPTG) at 20°C, cells were centrifuged at 8,000 × *g* for 15 min at 4°C; resuspended in 50 mM Tris [pH 8.0], 100 mM NaCl, 10 mM imidazole, and 1 mM β-mercaptoethanol (BME); lysed by sonication; and then centrifuged at 12,000 × *g* for 40 min at 4°C. The resulting supernatants were heated at 70°C for 10 min and centrifuged as above. Recombinant proteins were purified over Ni-NTA resin (Qiagen), washed with Buffer B (50 mM Tris [pH 8.0], 500 mM NaCl, 30 mM imidazole, and 1 mM BME) followed by Buffer C (50 mM Tris [pH 8.0], 200 mM NaCl, 30 mM imidazole, and 1 mM BME). Proteins were eluted with Buffer C containing 300 mM imidazole, followed by size exclusion chromatography over a Superdex 200 Increase 10/300 GL column (Cytiva) in Buffer C lacking imidazole.

### Pull down of *Pf*Trx^BamA/ECL4^ using IRS.

Sixty microliters of IRS or NRS was added to an equal volume of Protein G agarose beads (Thermo-Fisher) following which 150 ng of *Pf*Trx^BamA/ECL4^ or *Pf*Trx was added and then rotated overnight at room temperature (RT). After thorough washing with buffer consisting of 150 mM Tris (pH 7.5) and 1% Triton X-100, the beads were collected by centrifugation at 5,000 × *g* for 5 min. The beads were resuspended in 4× Laemmli Sample Buffer with 1 mM BME followed by boiling for 20 min. Following centrifugation at 10,000 × *g*, the supernatants were collected and stored at 4°C prior to SDS-PAGE and immunoblotting (see below).

### Immunization of rabbits with *Pf*Trx^TP0856/ECL2^ and *Pf*Trx^TP0856/ECL4^.

Two adult, male NZW rabbits were primed by four subcutaneous injections and two intramuscular injections with 100 μL and 50 μL PBS-TiterMax (1:1, vol/vol) respectively, containing a total of 200 μg of *Pf*Trx^TP0856/ECL2^ or *Pf*Trx^TP0856/ECL4^. Rabbits were boosted at 3, 6, and 9 weeks with the same volumes and amounts of protein in PBS-TiterMax (1:1, vol/vol) and exsanguinated 12 weeks postimmunization.

### Immunoblot analysis.

MARBLOT strips containing *Tp* lysates (Trinity Biotech) were blocked for 1 h with PBS, 5% nonfat dry milk and 0.1% Tween 20 and then probed overnight at 4°C with IRS or NRS (1:1,000). After washing with PBS containing 0.05% Tween 20 (PBST), the strips were incubated for 1 h at RT with horseradish peroxidase (HRP)-conjugated goat anti-rabbit IgG (1:30,000). Following washes with PBST, the immunoblot strips were developed on a single film using the SuperSignal West Pico chemiluminescent substrate.

To assess the reactivity of *Pf*Trx^BamA/ECL4^ with IRS and HSS, 400 ng of each protein and 20 ng of Tpp17 were resolved by SDS-PAGE using Any kD Mini-Protean TGX gels (Bio-Rad) and transferred to nitrocellulose membranes (0.45 μm) (Bio-Rad). The membranes were blocked for 1 h with PBS containing 5% nonfat dry milk and 0.1% Tween 20 and then probed overnight at 4°C with individual IRS, HSS, NRS, or normal human serum (NHS) (1:250). After being washed with PBST, membranes were incubated for 1 h at RT with HRP-conjugated goat anti-rabbit IgG or anti-human IgG (1:15,000). As additional controls, membranes with *Pf*Trx^BamA/ECL4^ and *Pf*Trx were probed with mouse anti-Avi-Tag Abs and rat anti-BamA ECL4 (12) (1:3,000 and 1:1,000, respectively) and HRP-conjugated goat anti-mouse Ig Abs and HRP-conjugated goat anti-rat Ig Abs (1:30,000). To verify immunoprecipitation of *Pf*Trx^BamA/ECL4^, eluates and supernatants were probed with mouse anti-Avi-Tag (1:3,000) followed by HRP-conjugated goat anti-mouse Ig Abs (1:30,000).

To assess the reactivity of TP0856 and TP0858 with IRS and HSS, 100 ng of each protein and 10 ng of Tpp17 were immunoblotted with 1:250 dilutions of each serum using the same conditions as above. To evaluate the reactivity of the TP0856 and TP0858 *Pf*Trx^ECL^ and *Pf*Trx^Hatch^ constructs with IRS, 400 ng of each protein, along with 400 ng of *Pf*Trx control, were immunoblotting using IRS (1:250) and HRP-conjugated goat anti-rabbit IgG (1:30,000).

To determine cross-reactivity between ECL2 and ECL4 of TP0856 and TP0858, 200 ng of TP0856 and TP0858 was immunoblotted as detailed above with polyclonal rabbit Abs generated against *Pf*Trx^TP0856/ECL2^ or *Pf*Trx^TP0856/ECL4^ (1:1,000) and HRP-conjugated anti-rabbit IgG (1:20,000).

### Reactivity of *Pf*Trx scaffolds with syphilitic sera by ELISA.

Clear Flat-Bottom Immuno Nonsterile 96 or 384-well plates (Thermo Scientific) were coated with streptavidin (SP) (Invitrogen) diluted in 0.1 M sodium bicarbonate at 200 ng/well and incubated overnight at 4°C. After washing, the plates were blocked with PBS buffer containing 15% goat serum, 0.005% Tween 20 and 0.05% sodium azide for 1 h at RT. Biotinylated *Pf*Trx^ECLs^ and *Pf*Trx^Hatch^ proteins were added at 200 ng/well in blocking buffer followed by 1 h of incubation at RT. After washing with PBS containing 0.1% Tween 20 (PBST), IRS or HSS were added in serial dilutions in PBS with 1% bovine serum albumin (BSA) followed by 1 h of incubation at RT. After washing with 0.1% PBST, HRP-conjugated goat anti-rabbit was added at a dilution of 1:10,000 followed by incubation for 1 h at RT. Plates were washed again with 0.1% PBST and developed with TMB single solution (Life Technologies). Reactions were stopped with 0.3 M HCl and read at 450-nm wavelength. The optical density readings of serial dilutions for each *Pf*Trx construct were used to calculate area under the curve (AUC). The AUC for *Pf*Trx alone was subtracted from the AUC of each *Pf*Trx construct.

### Determination of TP0856 and TP0858 sequences in *T. pallidum* clinical strains.

Described in detail in Text S1.

### Prediction of B-cell epitopes.

Predicted B-cell epitopes (BCEs) in TP0856 and TP0858 were identified using DiscoTope 2.0 (31), ElliPro (32), IEDB (33), and BC pred (34). Default settings were used for initial analyses. The BCE predictions were mapped onto one-dimensional depictions of the TP0856 and TP0858 structures using previously reported boundaries for the hatches and ECLs (6). PDBs of the TP0856 and TP0858 3D models (downloadable from https://drive.google.com/file/d/1EurEnlwAiqtsUm8t-jC3Xuz5e7nV45mT/view?usp=sharing&export=download) (6) were used to identify predicted conformational epitopes with DiscoTope 2.0 and ElliPro.

### Identification of specific B cells by flow cytometry.

Ficoll-Hypaque density gradient centrifugation was used to isolate PBMCs from rabbit blood; PBMCs cryopreserved in liquid nitrogen were used for flow cytometry experiments. The following rabbit-specific Abs were used (Table S2): IgM-FITC, IgA-DyLight594, and IgG-PE. *In vivo* biotinylated *Pf*Trx ECL and Hatch constructs, Tpp17, and OspC were conjugated with SP-AF405 and SP-AF647, whereas *Pf*Trx alone was conjugated with SP-APC-Cy7. PBMCs were stained at 4°C for 15 min, washed and resuspended with fixation buffer (BD Biosciences). Lymphocytes were gated from all cells and doublets were excluded in FSC-H and -W, and SSC-H and -W plots. IgM and IgA double-negative cells were then gated, and from this gate IgG^+^ cells were identified. Cells binding SP-APC-Cy7-*Pf*Trx were excluded from IgG^+^ cells. From *Pf*Trx^Neg^ cells, double-positive cells for SP-AF405 and SP-AF647 conjugated with *Pf*Trx^ECLs^ or *Pf*Trx^Hatch^ were identified as antigen-specific IgG^+^ cells. Tpp17- and OspC-specific cells were gated directly from IgG^+^ cells. Flow cytometry was performed on a LSR II flow cytometer (BD Biosciences) and data analysis was conducted using FlowJo Version 10.7.1 software (Tree Star).

### Statistical analysis.

The means of the AUC from ELISA dilution curves for each construct (*Pf*Trx^TP0856/ECLs^, *Pf*Trx^TP0856/Hatch^, *Pf*Trx^TP0858/ECLs^, and *Pf*Trx^TP0858/Hatch^) were compared to determine statistical significance by one-way ANOVA with Bonferroni's correction for multiple comparisons using PRISM 9.0 (GraphPad Software).
