# Supplementary material for: Extracellular Loops of the Treponema pallidum FadL Orthologs TP0856 and TP0858 Elicit IgG Antibodies and IgG+-Specific B-Cells in the Rabbit Model of Experimental Syphilis
Source: mBio. 2022 Jul 12;13(4):e01639-22. doi: 10.1128/mbio.01639-22 (PMC9426418; doi:10.1128/mbio.01639-22)
Supplement: Text S1 [file mbio.01639-22-s0001.docx]

**Extracellular loops of the Treponema pallidum FadL orthologs TP0856 and TP0858 elicit IgG antibodies and IgG^+^-specific B-cells in the rabbit model of experimental syphilis**

**Supplemental materials**

**SUPPLEMENTAL MATERIALS AND METHODS**

**Determination of TP0856 and TP0858 sequences in *T. pallidum* clinical strains**

*T. pallidum* genomic DNA was extracted from two skin biopsies and a lesion swab obtained from three adult HIV-seronegative patients with secondary syphilis (SS) and shipped to the University of North Carolina at Chapel Hill (UNC) for whole-genome sequencing. *T. pallidum* DNA and total DNA were quantified using quantitative real-time PCR (qPCR) targeting the *T. pallidum* DNA polymerase I (*polA*) gene as previously described (1) and a Qubit 3 fluorometer (Thermo Fischer Scientific, Waltham, MA) using dsDNA HS2 reagents, respectively. *T. pallidum* DNA was enriched using Sure Select XT Low Input (Agilent Technologies, Santa Clara, CA) custom 120-nucleotide RNA oligonucleotide baits as described previously (2), with the following modifications: improved bait design and without pooled parallel whole-genome amplification prior to bait capture. In brief, we designed a new set of baits by tiling baits contiguously (1x) across the complete SS14 (CP004011.1) and Nichols (CP004010.2) genome assemblies, and by stacking baits (5x) across putative outer membrane protein and phylogenetically informative gene targets of interest. Sequences from these targets were extracted from the 74 *T. pallidum* genomic sequences included in our prior analysis (2). Duplicate baits with ≥ 95% sequence identity were removed. The remaining baits were screened for complementarity to human and New Zealand white rabbit genomes; none had cross-hybridization potential. After library preparation, samples with similar input *T. pallidum* DNA concentrations were pooled into up to 16-sample pools prior to bait capture. Samples were sequenced at the UNC High Throughput Sequencing Facility using the MiSeq (Illumina, San Diego, CA) platform with 150bp paired-end reads.

Sequencing reads were processed using a conservative bioinformatic pipeline as previously described with minor modifications (2), available at https://doi.org/10.5281/zenodo.5773174. In summary, raw reads were first trimmed using *trimmomatic.* Trimmed sequences that mapped to a combined reference containing the human genome and animal, plant, and fungal ribosomal sequences using *bbmap* were removed. Clean, paired-end reads were then aligned to the SS14 (CP004011.1) or Nichols strain (CP004010.2) reference genomes based upon clade assignments using *bwa. PICARD* and *GATK* tools were used to remove duplicate reads and realign indels, respectively. Reads were subjected further to stringent filters, including removal of low-quality mapping and multi-mapping reads, as previously described by Grillova *et al*., (3) and Chen *et al*., (2). Variant calling was performed using the *GATK* HaplotypeCaller utility, and joint genotyping was performed using the GenotypeVCFs module with hard filtering as previously described (2). Base calls were made at loci covered by at least 3 unique, filtered reads, and sequences for the TP0856 and TP0858 loci extracted for further analysis.

To close a gap in the *tp0858* sequence for one sample, we first performed selective whole-genome amplification (sWGA) using primers and conditions described by Thurlow *et al*., (4) to overcome low *T. pallidum* DNA concentration. The amplified sWGA product was cleaned up using a 1.8x ratio of Ampure XP beads (Beckman Coulter, Pasadena, CA). The affected region was amplified using a nested PCR assay using oligonucleotide sequences (**Table S3**) designed in Primer3PLUS (5). PCR was performed using a 50µL total reaction volume comprising 1 µL DNA template (sWGA product), 1.25U AmpliTaq DNA polymerase (Thermo Fisher), 1X Buffer I, 0.2 µM primers, and 0.2 mM each dNTP. The reaction was denatured at 95°C for 2 min, and amplified for 40 cycles of 95°C for 2 min, 55°C for 30 sec, and 72°C for 45 sec, followed by a final extension at 72°C for 5 min. Sanger sequencing was performed at Eton Biosciences (Research Triangle, NC). Chromatograms were resolved and sequences trimmed using Geneious Prime software (version 2020.2.2, Biomatters, San Diego, CA). Illumina and Sanger sequencing data will be deposited in the Sequence Read Archive and GenBank, respectively (BioProject Accession number: PRJNA815321). Protein sequences for TP0856 and TP0858 were aligned using Clustal Omega in MacVector, with the Nichols strain sequences as the references.

**REFERENCES**

1. Marfin AA, Liu H, Sutton MY, Steiner B, Pillay A, Markowitz LE. 2001. Amplification of the DNA polymerase I gene of *Treponema pallidum* from whole blood of persons with syphilis. DiagnMicrobiolInfectDis 40:163-166.

2. Chen W, Smajs D, Hu Y, Ke W, Pospisilova P, Hawley KL, Caimano MJ, Radolf JD, Sena A, Tucker JD, Yang B, Juliano JJ, Zheng H, Parr JB. 2021. Analysis of *Treponema pallidum* Strains From China Using Improved Methods for Whole-Genome Sequencing From Primary Syphilis Chancres. J Infect Dis 223:848-853.

3. Grillova L, Oppelt J, Mikalova L, Novakova M, Giacani L, Niesnerova A, Noda AA, Mechaly AE, Pospisilova P, Cejkova D, Grange PA, Dupin N, Strnadel R, Chen M, Denham I, Arora N, Picardeau M, Weston C, Forsyth RA, Smajs D. 2019. Directly Sequenced Genomes of Contemporary Strains of Syphilis Reveal Recombination-Driven Diversity in Genes Encoding Predicted Surface-Exposed Antigens. Front Microbiol 10:1691.

4. Thurlow CM, Joseph SJ, Ganova-Raeva L, Katz SS, Pereira L, Chen C, Debra A, Vilfort K, Workowski K, Cohen SE, Reno H, Sun Y, Burroughs M, Sheth M, Chi KH, Danavall D, Philip SS, Cao W, Kersh EN, Pillay A. 2022. Selective Whole-Genome Amplification as a Tool to Enrich Specimens with Low *Treponema pallidum* Genomic DNA Copies for Whole-Genome Sequencing. mSphere:e0000922.

5. Untergasser A, Nijveen H, Rao X, Bisseling T, Geurts R, Leunissen JA. 2007. Primer3Plus, an enhanced web interface to Primer3. Nucleic Acids Res 35:W71-4.
