# Supplementary figures and images for: Extracellular Loops of the Treponema pallidum FadL Orthologs TP0856 and TP0858 Elicit IgG Antibodies and IgG+-Specific B-Cells in the Rabbit Model of Experimental Syphilis
Source: mBio. 2022 Jul 12;13(4):e01639-22. doi: 10.1128/mbio.01639-22 (PMC9426418; doi:10.1128/mbio.01639-22)

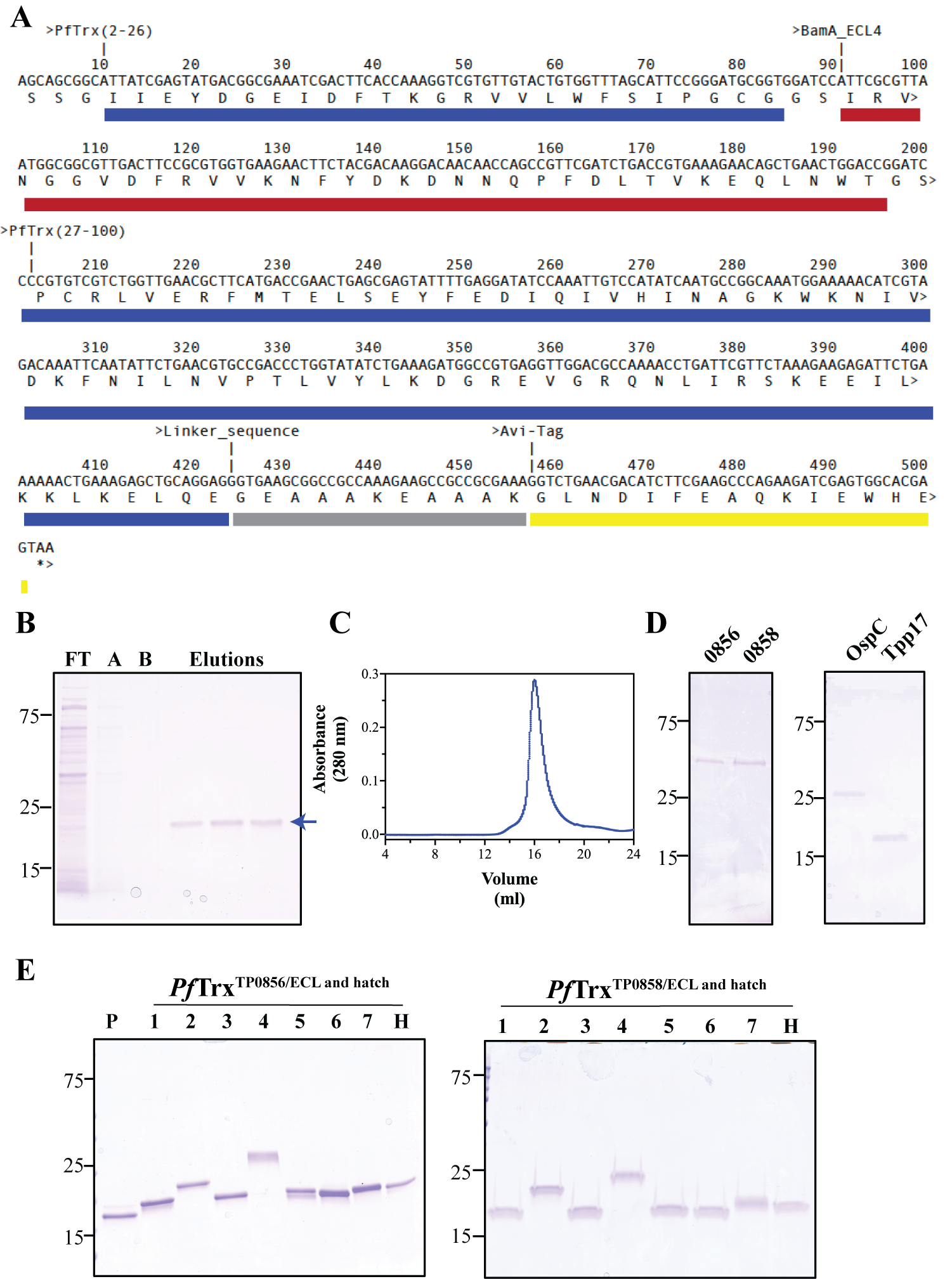

Supplement: FIG S1 [file mbio.01639-22-sf001.tif]
