# Supplementary material for: Extracellular Loops of the Treponema pallidum FadL Orthologs TP0856 and TP0858 Elicit IgG Antibodies and IgG+-Specific B-Cells in the Rabbit Model of Experimental Syphilis
Source: mBio. 2022 Jul 12;13(4):e01639-22. doi: 10.1128/mbio.01639-22 (PMC9426418; doi:10.1128/mbio.01639-22)
Supplement: TABLE S1 [file mbio.01639-22-st001.docx]

| **Table S1.** Summary of area under the curve (AUC) values from ELISA dilution curves. | | | | | | | | |
| --- | --- | --- | --- | --- | --- | --- | --- | --- |
|  |  |  |  |  |  |  |  |  |
| ***Pf*Trx^TP0856^** | **ECL1** | **ECL2** | **ECL3** | **ECL4** | **ECL5** | **ECL6** | **ECL7** | **Hatch** |
| IRS 112 | -0.0626 | **1.0926** | **1.6287** | **1.8635** | 0.8196 | 0.6229 | 0.3252 | **1.1851** |
| IRS 113 | -0.0042 | 0.0486 | 0.1636 | **2.2226** | 0.0653 | 0.0596 | 0.2964 | 0.2441 |
| IRS 114 | 0.0081 | 0.5785 | 0.3491 | **1.6576** | 0.2762 | 0.1198 | 0.2076 | 0.3833 |
|  |  |  |  |  |  |  |  |  |
| ***Pf*Trx^TP0858^** | **ECL1** | **ECL2** | **ECL3** | **ECL4** | **ECL5** | **ECL6** | **ECL7** | **Hatch** |
| IRS 112 | 0.6646 | 0.9924 | 0.5276 | **1.8195** | 0.4548 | 0.3684 | 0.4548 | 0.0170 |
| IRS 113 | 0.0865 | -0.0016 | 0.0151 | **1.5711** | 0.2590 | -0.0042 | 0.2590 | -0.0805 |
| IRS 114 | -0.1216 | 1.3724 | -0.1363 | **1.2181** | 0.1155 | -0.1365 | 0.1155 | -0.1957 |
