# Supplementary material for: Extracellular Loops of the Treponema pallidum FadL Orthologs TP0856 and TP0858 Elicit IgG Antibodies and IgG+-Specific B-Cells in the Rabbit Model of Experimental Syphilis
Source: mBio. 2022 Jul 12;13(4):e01639-22. doi: 10.1128/mbio.01639-22 (PMC9426418; doi:10.1128/mbio.01639-22)
Supplement: TABLE S2 [file mbio.01639-22-st002.docx]

| **Table S2.** Summary of antibody reagents used for identification of IgG+ antigen-specific rabbit B-cells. | | | | |
| --- | --- | --- | --- | --- |
|  |  |  |  |  |
| **Marker/Reagent** | **Cell type** | **Fluorophore** | **Supplier** | **Catalog #** |
| Anti-rabbit IgM | B-cells | FITC | Bio-Rad | 402002 |
| Anti-rabbit IgA | B-cells | DyLight 594 | Bethyl | A120-109D4 |
| Anti-rabbit IgG | Memory B-cells | PE | Southern Biotech | 4030-09 |
| Antigen | *Pf*Trx specific B-cells | SP-APC/Cyanine7 | BioLegend | 405208 |
| Antigen | *T. pallidum* specific B-cells | SP-Alexa Fluor 647 | BioLegend | 405237 |
| Antigen | *T. pallidum* specific B-cells | SP-Alexa Fluor 405 | Invitrogen | 2160388 |
