# Supplementary material for: Extracellular Loops of the Treponema pallidum FadL Orthologs TP0856 and TP0858 Elicit IgG Antibodies and IgG+-Specific B-Cells in the Rabbit Model of Experimental Syphilis
Source: mBio. 2022 Jul 12;13(4):e01639-22. doi: 10.1128/mbio.01639-22 (PMC9426418; doi:10.1128/mbio.01639-22)
Supplement: TABLE S3 [file mbio.01639-22-st003.docx]

| **Protein** | **Name** | **Primer name** | **Description** | **Oligo Sequence** | **Amplicon Sequence** | **Amino Acid Sequence** |
| --- | --- | --- | --- | --- | --- | --- |
| **BamA** | *Pf*Trx^BamA/ECL4^ | *Pf*Trx^BamA/ECL4^ –FW | Amplification of BamA ECL4 ^568-602^ | CGCGCGGCAGCCATATGAGCAGCGGCATTATCGAG | ATTCGCGTTAATGGCGGCGTTGACTTCCGCGTGGTGAAGAACTTCTACGACAAGGACAACAACCAGCCGTTCGATCTGACCGTGAAAGAACAGCTGAACTGGACC | IRVNGGVDFRVVKNFYDKDNNQPFDLTVKEQLNWT |
|  |  | *Pf*Trx^BamA/ECL4^ –RV |  | GGTGGTGGTGCTCGAGTTACTGTGCCACTCGATC |  |  |
| **TP0856** | TP0856 | Full-length FadL TP0856 –FW | Amplification of TP0856 | AGCCATATGGCTAGCAGCGAGGCGGCGGCGAAG |  |  |
|  |  | Full-length FadL TP0856 –RV |  | GTGGTGGTGGTGGTGTTATTTACGACCCAGCAGGATG |  |  |
|  | *Pf*Trx^TP0856/ECL1^ | *Pf*Trx^TP0856/ECL1^–FW | Amplification of TP0856 ECL1^91-107^ | TAACAACAGCCACGCGGAGACCCTGAGCTACGTTGGATCCCCGTGTCGTCTGGT | GCGCACACCGTGGGCTTTAACAACAGCCACGCGGAGACCCTGAGCTACGTT | AHTVGFNNSHAETLSYV |
|  |  | *Pf*Trx^TP0856/ECL1^–RV |  | GCGTGGCTGTTGTTAAAGCCCACGGTGTGCGCGGATCCACCGCATCCCGG |  |  |
|  | *Pf*Trx^TP0856/ECL2^ | *Pf*Trx^TP0856/ECL2^–5' | Amplification of TP0856 ECL2^119-156^ | GGGATGCGGTGGATCCATGCGTATGTTCTTTCCGGAAAGC | ATGCGTATGTTCTTTCCGGAAAGCGGTTTCAACTTTAGCCCGAGCACCGGTCCGGTGTGCACCCCGGCGAGCAACCCGATCAAGAAACTGGGTGGCCTGGGTATTGTTAACTTC | MRMFFPESGFNFSPSTGPVCTPASNPIKKLGGLGIVNF |
|  |  | *Pf*Trx^TP0856/ECL2^–3' |  | GACGACACGGGGATCCGAAGTTAACAATACCCAGGCCACCC |  |  |
|  | *Pf*Trx^TP0856/ECL3^ | *Pf*Trx^TP0856/ECL3^–FW | Amplification of TP0856 ECL3^172-183^ | CAGACCCTGCGCATCACGGAAACCGGATCCACCGCATCCCGG | GGTTTCCGTGATGCGCAGGGTCTGACCCACCTGAGC | WVGNVAKFFSSAEPNM |
|  |  | *Pf*Trx^TP0856/ECL3^–RV |  | GATGCGCAGGGTCTGACCCACCTGAGCGGATCCCCGTGTCGTCTGGT |  |  |
|  | *Pf*Trx^TP0856/ECL4^ | *Pf*Trx^TP0856/ECL4^–5' | Amplification of TP0856 ECL4^222-284^ | GGGATGCGGTGGATCCCTGCCGGGCAGCCCG | CTGCCGGGCAGCCCGTTTGTTCTGTGCCGTGCGACCGGTGAACAGTGCTGCAAAACCTGCAGCGGCCGTTGCACCGGTGTTGGCACCTGCTGCAACGGCGAGAAGCCGTGCTGCAAAGACTGCGATTGCAACTGCCCGTGCCAGGATGAAGCGACCCCGGGTAGCCCGCACGCGACCGATACCATGCTG | LPGSPFVLCRATGEQCCKTCSGRCTGVGTCCNGEKPCCKDCDCNCPCQDEATPGSPHATDTML |
|  |  | *Pf*Trx^TP0856/ECL4^–3' |  | GACGACACGGGGATCCCAGCATGGTATCGGTCGCGT |  |  |
|  | *Pf*Trx^TP0856/ECL5^ | *Pf*Trx^TP0856/ECL5^–FW | Amplification of TP0856 ECL5^304-318^ | CAACCTGCAGGTGGACCACCTGTGGAAGGGATCCCCGTGTCGTCTGGT | ACCCGTGTGAACGTTAGCAACCTGCAGGTGGACCACCTGTGGAAG | TRVNVSNLQVDHLWK |
|  |  | *Pf*Trx^TP0856/ECL5^–RV |  | TCCACCTGCAGGTTGCTAACGTTCACACGGGTGGATCCACCGCATCCCGG |  |  |
|  | *Pf*Trx^TP0856/ECL6^ | *Pf*Trx^TP0856/ECL6^–FW | Amplification of TP0856 ECL6^342-352^ | AGTGCGTGCGGGTGTTGGATCCCCGTGTCGTCTGGT | ACCCGTGTGAACGTTAGCAACCTGCAGGTGGACCACCTGTGGAAG | VNANGKVRAGV |
|  |  | *Pf*Trx^TP0856/ECL6^–RV |  | ACACCCGCACGCACTTTGCCGTTCGCGTTAACGGATCCACCGCATCCCGG |  |  |
|  | *Pf*Trx^TP0856/ECL7^ | *Pf*Trx^TP0856/ECL7^–FW | Amplification of TP0856 ECL7^370-382^ | ACAGCAAGGTACCCCGCACAACGGATCCCCGTGTCGTCTGGT | GACAGCACCGGTGATGAACAGCAAGGTACCCCGCACAAC | DSTGDEQQGTPHN |
|  |  | *Pf*Trx^TP0856/ECL7^–RV |  | GGGGTACCTTGCTGTTCATCACCGGTGCTGTCGGATCCACCGCATCCCGG |  |  |
|  | *Pf*Trx^TP0856/Hatch^ | *Pf*Trx^TP0856/Hatch^–FW | Amplification of TP0856 Hatch^29-40^ | GCGGCGGCGAAGACCCGTAGCAAAATGGGATCCCCGTGTCGTCTGGT | AGCAGCGAGGCGGCGGCGAAGACCCGTAGCAAAATG | SSEAAAKTRSKM |
|  |  | *Pf*Trx^TP0856/Hatch^–RV |  | GGTCTTCGCCGCCGCCTCGCTGCTGGATCCACCGCATCCCGG |  |  |
| **TP0858** | TP0858 | Full-length FadL TP0858 –FW | Amplification of TP0858 | AGCCATATGGCTAGCGCGGCGGCGAAGCCGAAGAAAGG |  |  |
|  |  | Full-length FadL TP0858 –RV |  | GTGGTGGTGGTGGTGTTAGTTACGACCCAGCAGAATCGC |  |  |
|  | *Pf*Trx^TP0858/ECL1^ | *Pf*Trx^TP0858/ECL1^–FW | Amplification of TP0858 ECL1^103-114^ | GCTGCCGAAACCGGTGGTGTGAAAGGATCCACCGCATCCCGG | TTTCACACCACCGGTTTCGGCAGCTTTCACGCGGAA | FHTTGFGSFHAE |
|  |  | *Pf*Trx^TP0858/ECL1^–RV |  | ACCGGTTTCGGCAGCTTTCACGCGGAAGGATCCCCGTGTCGTCTGGT |  |  |
|  | *Pf*Trx^TP0858/ECL2^ | *Pf*Trx^TP0858/ECL2^–5' | Amplification of TP0858 ECL2^133-162^ | GGGATGCGGTGGATCCATGCGTATGTTCTTTCCGGAAAGCG | ATGTTCTTTCCGGAAAGCGGTTTCGACTTTAGCACCACCACCGAGCCGGTTTGCACCCCGGCGAGCAACCCGATCAAGCAACGTGGTGCG | MFFPESGFDFSTTTEPVCTPASNPIKQRGA |
|  |  | *Pf*Trx^TP0858/ECL2^–3' |  | GACGACACGGGGATCCGATGCCAATCGCACCACGTTG |  |  |
|  | *Pf*Trx^TP0858/ECL3^ | *Pf*Trx^TP0858/ECL3^–FW | Amplification of TP0858 ECL3^184-195^ | CAGACCCTGCGCGTCACGAAAGCCGGATCCACCGCATCCCGG | GGCTTTCGTGACGCGCAGGGTCTGCAACACACCAGC | GFRDAQGLQHTS |
|  |  | *Pf*Trx^TP0858/ECL3^–RV |  | GACGCGCAGGGTCTGCAACACACCAGCGGATCCCCGTGTCGTCTGGT |  |  |
|  | *Pf*Trx^TP0858/ECL4^ | *Pf*Trx^TP0858/ECL4^–5' | Amplification of TP0858 ECL4^224-289^ | GGGATGCGGTGGATCCGCGGCGACCAACCTGGG | GCGGCGACCAACCTGGGTCTGACCGTGAAAGTTAGCGACAAAATTGAGAACTGCACCAGCACCTGCGAAAAGTGCGGCTGCTGCAAAGAGCGTTGCTGCTGCAACGGTAAGAAAGCGTGCTGCAAGGACTGCGATTGCAACTGCCCGTGCCAAGACTGCAACGATAAAGGCACCGTGCACGCGACCGATACCATGCTG | AATNLGLTVKVSDKIENCTSTCEKCGCCKERCCCNGKKACCKDCDCNCPCQDCNDKGTVHATDTML |
|  |  | *Pf*Trx^TP0858/ECL4^–3' |  | GACGACACGGGGATCCCAGCATGGTATCGGTCGCGT |  |  |
|  | *Pf*Trx^TP0858/ECL5^ | *Pf*Trx^TP0858/ECL5^–FW | Amplification of TP0858 ECL5^309-331^ | GCTCTTCGCGTCGCTGCTCGCCAGGGTCTGAACGTTCATGCTGGTGGATCCACCGCATCCCGGAATGCTAAA | ACCAGCATGAACGTTCAGACCCTGGCGAGCAGCGACGCGAAGAGCCTGTACCAAAACCTGGCGTATAGC | TSMNVQTLASSDAKSLYQNLAYS |
|  |  | *Pf*Trx^TP0858/ECL5^–RV |  | AGCGACGCGAAGAGCCTGTACCAAAACCTGGCGTATAGCGGATCCCCGTGTCGTCTGGTTGAACG |  |  |
|  | *Pf*Trx^TP0858/ECL6^ | *Pf*Trx^TP0858/ECL6^–FW | Amplification of TP0858 ECL6^347-358^ | CGCCTTGTGGTTAATACGAAAGCTGGATCCACCGCATCCCGG | AGCTTTCGTATTAACCACAAGGCGAACATGCGTGTG | SFRINHKANMRV |
|  |  | *Pf*Trx^TP0858/ECL6^–RV |  | ATTAACCACAAGGCGAACATGCGTGTGGGATCCCCGTGTCGTCTGGT |  |  |
|  | *Pf*Trx^TP0858/ECL7^ | *Pf*Trx^TP0858/ECL7^–FW | Amplification of TP0858 ECL7^376-396^ | GCAGCCGCTACCGCTGCTGATATCGCTCACGTCGCAACGGGATCCACCGCATCCCGG | CGTTGCGACGTGAGCGATATCAGCAGCGGTAGCGGCTGCACCGGTGCGAAAGCGAGCCACTAT | RCDVSDISSGSGCTGAKASHY |
|  |  | *Pf*Trx^TP0858/ECL7^–RV |  | AGCGGTAGCGGCTGCACCGGTGCGAAAGCGAGCCACTATGGATCCCCGTGTCGTCTGGT |  |  |
|  | *Pf*Trx^TP0858/Hatch^ | *Pf*Trx^TP0858/Hatch^–FW | Amplification of TP0858 Hatch^43-60^ | GTTTTTGCATTTGACCTTTCTTCGGCTTCGCCGCCGCGGATCCACCGCATCCCGGAATGCTAAA | GCGGCGGCGAAGCCGAAGAAAGGTCAAATGCAAAAACTGCGTCAACGTCCGGTG | AATAAAKPKKGQMQKLRQRPV |
|  |  | *Pf*Trx^TP0858/Hatch^–RV |  | GGTCAAATGCAAAAACTGCGTCAACGTCCGGTGGGATCCCCGTGTCGTCTGGTTGAACG |  |  |
| **Tpp17** | Tpp17 | Tpp17_P28BIOH –FW | Amplification of Tpp17 | GCTGGAGGTTCAGGTTGCACAACCGTGTGTCCG |  |  |
|  |  | Tpp17_P28BIOH –RV |  | ATGACCACTTCCACCTTTCTTTGTTTTTTTGAGCACGTAAAACGGC |  |  |
| **OspC** | *Bb* OspC | OspC-vec-FW | Amplification of His-Avitag vector backbone | AGCCGCCGCGAAAGGTCT | TGTAATAATTCAGGGAAAGATGGGAATACATCTGCAAATTCTGCTGATGAGTCTGTTAAAGGGCCTAATCTTACAGAAATAAGTAAAAAAATTACGGATTCTAATGCGGTTTTACTTGCTGTGAAAGAGGTTGAAGCGTTGCTGTCATCTATAGATGAAATTGCTGCTAAAGCTATTGGTAAAAAAATACACCAAAATAATGGTTTGGATACCGAAAATAATCACAATGGATCATTGTTAGCGGGAGCTTATGCAATATCAACCCTAATAAAACAAAAATTAGATGGATTGAAAAATGAAGGATTAAAGGAAAAAATTGATGCGGCTAAGAAATGTTCTGAAACATTTACTAATAAATTAAAAGAAAAACACACAGATCTTGGTAAAGAAGGTGTTACTGATGCTGATGCAAAAGAAGCCATTTTAAAAACAAATGGTACTAAAACTAAAGGTGCTGAAGAACTTGGAAAATTATTTGAATCAGTAGAGGTCTTGTCAAAAGCAGCTAAAGAGATGCTTGCTAATTCAGTTAAAGAGCTTACAAGCCCTGTTGTGGCAGAAAGTCCAAAAAAACC | CNNSGKDGNTSANSADESVKGPNLTEISKKITDSNAVLLAVKEVEALLSSIDEIAAKAIGKKIHQNNGLDTENNHNGSLLAGAYAISTLIKQKLDGLKNEGLKEKIDAAKKCSETFTNKLKEKHTDLGKEGVTDADAKEAILKTNGTKTKGAEELGKLFESVEVLSKAAKEMLANSVKELTSPVVAESPKKP |
|  |  | OspC-vec-RV |  | GCCGCTGCTCATATGGCTG |  |  |
|  |  | OspC-ins-FW | Amplification of OspC | CATATGAGCAGCGGCTGTAATAATTCAGGGAAAGATGGGA |  |  |
|  |  | OspC-ins-RV |  | CCTTTCGCGGCGGCTAGGTTTTTTTGGACTTTCTGCC |  |  |
| **gene** | **Name** | **Primer name** | **Description** | **Oligo Sequence (5' --> 3')** |  |  |
| ***tp0858*** | *tp0858* | 858g_F_outer | Nested PCR | GCGGGGCAATTGGAATCATC |  |  |
|  |  | 858g_R_outer |  | ACTCCCACTCGCATGTTAGC |  |  |
|  |  | 858g_F_inner |  | CCTCTGAAGAGCCCAACCTG |  |  |
|  |  | 858g_R_inner |  | GAAGGGATCAAACATGGCGC |  |  |
