# Supplementary material for: Extracellular Loops of the Treponema pallidum FadL Orthologs TP0856 and TP0858 Elicit IgG Antibodies and IgG+-Specific B-Cells in the Rabbit Model of Experimental Syphilis
Source: mBio. 2022 Jul 12;13(4):e01639-22. doi: 10.1128/mbio.01639-22 (PMC9426418; doi:10.1128/mbio.01639-22)
Supplement: FIG S2 [file mbio.01639-22-sf002.pdf]

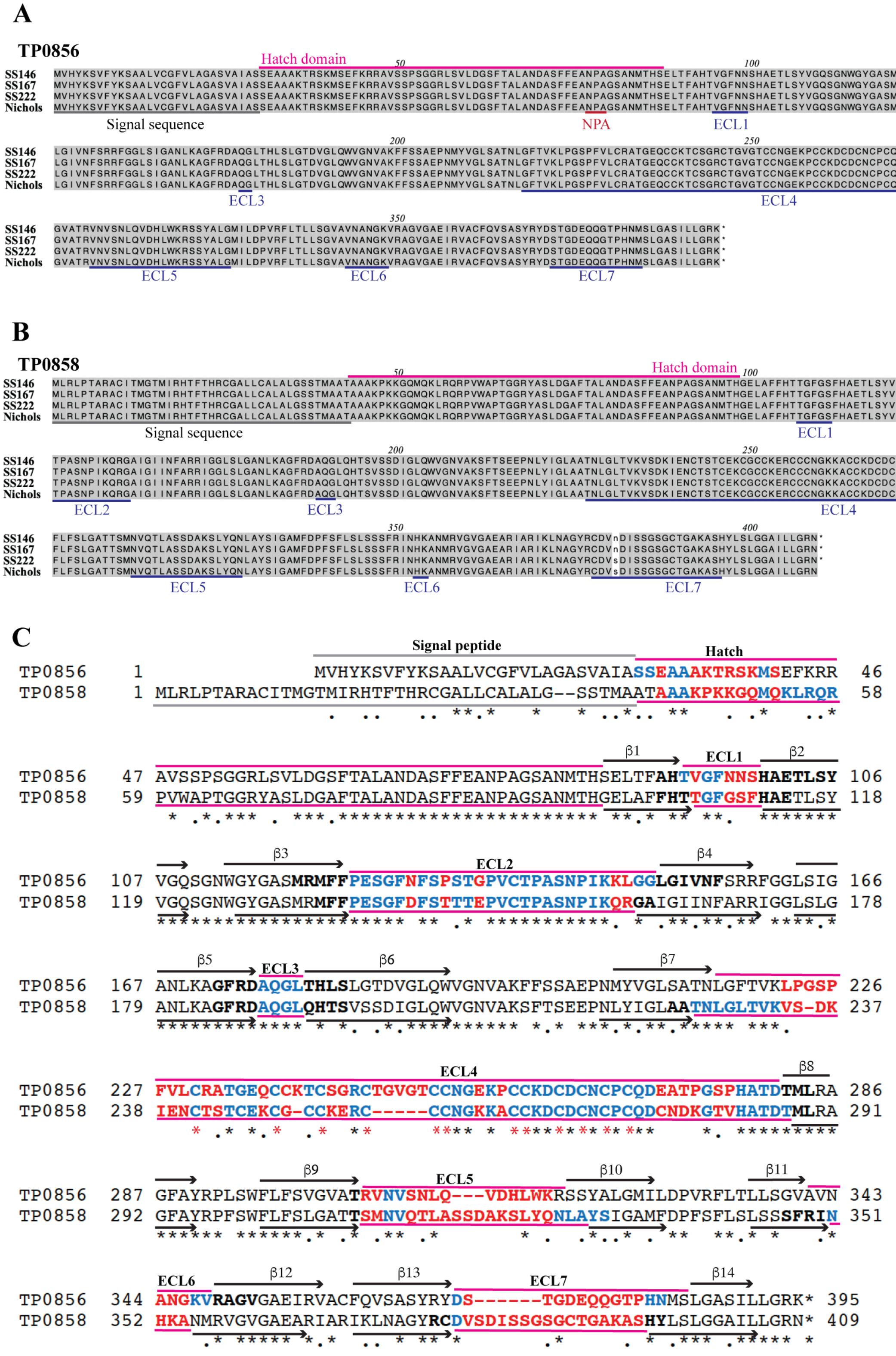
A
TP0856
Hatch domain
SS146
SS167
SS222
Nichols
Signal sequence
NPA
ECL1
ECL2
ECL3
ECL4
ECL5
ECL6
ECL7
B
TP0858
Hatch domain
SS146
SS167
SS222
Nichols
Signal sequence
ECL1
ECL2
ECL3
ECL4
ECL5
ECL6
ECL7
C
Signal peptide
Hatch
TP0856 1 MVHYKSVFYKSAALVCGFVLAGASVAIASSEAAAKTRSKMSEFKRR 46
TP0858 1 MLRLPTARACITMGTMIRHTFTHRCGALLCALALG--SSTMAATAAAKPKKGQMQKLRQR 58
β1
ECL1
β2
TP0856 47 AVSSPSGGRLSVLDGSFTALANDASFFEANPAGSANMTHSELTFAHTVGFNNSHAETLSY 106
TP0858 59 PVWAPTGGRYASLDGAFTALANDASFFEANPAGSANMTHGELAFFHTTGFGSFHAETLSY 118
β3
ECL2
β4
TP0856 107 VGQSGNWGYGASMRMFFPESGFNFSPSTGPVCTPASNPIKKLGGLGIVNFSRRFGGLSIG 166
TP0858 119 VGQSGNWGYGASMRMFFPESGFDFSTTTEPVCTPASNPIKQRGAIGIINFARRIGGLSLG 178
β5
ECL3
β6
β7
TP0856 167 ANLKAGFRDAQGLTHLSLGTDVGLQWVGNVAKFFSSAEPNMYVGLSATNLGFTVKLPGSP 226
TP0858 179 ANLKAGFRDAQGLQHTSVSSDIGLQWVGNVAKSFTSEEPNLYIGLAATNLGLTVKVS-DK 237
ECL4
β8
TP0856 227 FVLCRATGEQCCKTCSGRCTGVGTCCNGEKPCCKDCDCNCPCQDEATPGSPHATDTMLRA 286
TP0858 238 IENCTSTCEKCG-CCKERC-----CCNGKKACCKDCDCNCPCQDCNDKGTVHATDTMLRA 291
β9
ECL5
β10
β11
TP0856 287 GFAYRPLSWFLFSVGVATRVNVSNLQ---VDHLWKRSSYALGMILDPVRFLTLLSGVAVN 343
TP0858 292 GFAYRPFSWFLFSLGATTSMNVQTLASSDAKSLYQNLAYSIGAMFDPFSFLSLSSSFRIN 351
ECL6
β12
β13
ECL7
β14
TP0856 344 ANGKVRAGVGAEIRVACFQVSASYRYDS------TGDEQQGTPHNMSLGASILLGRK* 395
TP0858 352 HKANMRVGVGAEARIARIKLNAGYRCDVSDISSGSGCTGAKASHYLSLGGAILLGRN* 409
